# Serum cystatin C and cognitive function in midlife: results from the Hanzhong Adolescent Hypertension Study

**DOI:** 10.3389/fneur.2026.1739512

**Published:** 2026-02-10

**Authors:** Guilin Hu, Tongshuai Guo, Dan Wang, Ziyue Man, Mingfei Du, Wenling Zheng, Mingke Chang, Teng Zhang, Shenghao Zuo, Chenyang Liu, Ruiyu Wang, Chao Chu, Yu Yan, Yang Wang, Jianjun Mu

**Affiliations:** 1 Department of Cardiovascular Medicine, First Affiliated Hospital of Xi’an Jiaotong University, Xi’an, China; 2 Key Laboratory of Molecular Cardiology of Shaanxi Province, Xi’an, China; 3 Department of Geriatric-Cardiovascular Medicine, First Affiliated Hospital of Xi'an Jiaotong University, Xi’an, China

**Keywords:** albuminuria, cognitive impairment, cystatin C, hypertension, MMSE

## Abstract

**Background:**

Cystatin C (CysC), a low-molecular-weight protein, is widely used as a biomarker of renal function. The relationship between CysC and cognitive impairment remains controversial. This study aimed to investigate the association between CysC and cognitive impairment in the Hanzhong Adolescent Hypertension Study.

**Methods:**

A total of 2,347 participants completed the Mini-Mental State Examination (MMSE) in 2023, after excluding the participants missing data, 1929 participants were included. Multivariable logistic regression was used to assess the cross-sectional association between serum CysC levels and cognitive impairment. Subgroup and interaction analyses were performed to examine effect modification by albuminuria status. The nonlinear relationship was explored using restricted cubic splines (RCS).

**Results:**

Cognitive impairment was identified in 149 participants (7.72%). Each 1-SD increase in CysC levels was significantly associated with 49% higher odds of cognitive impairment after full adjustment [odds ratio (OR) = 1.49, 95% confidence interval (CI): 1.24–1.79, *p* < 0.001]. Compared with participants in the lowest CysC quartile, those in the highest quartile had significantly higher odds of cognitive impairment (OR = 1.79, 95% CI = 1.09–2.98, *p* = 0.022). The association was stronger in participants without albuminuria (OR per SD = 1.48, 95% CI: 1.22–1.80, *p* < 0.001) but absent in those with albuminuria (OR per SD = 1.00, 95% CI: 0.73–1.25, *p* = 0.981), with a significant interaction when CysC was modeled continuously (*p* for interaction = 0.028). A linear and positive association was observed between cystatin C levels and the prevalence of cognitive impairment (*p* for linearity < 0.001). This association remained significant in the non-albuminuria subgroup.

**Conclusion:**

Elevated CysC is associated with cognitive impairment assessed by MMSE in this midlife natural population cohort. This association is stronger in participants without albuminuria.

## 1 Introduction

Dementia, a condition secondary to neurodegenerative and psychiatric diseases, represents a growing global health challenge that significantly impairs quality of life (1, 2). Its prevalence is increasing by approximately 10 million cases annually. Cognitive impairment is widely recognized as a preclinical and transitional stage of dementia (3), primarily characterized by declines in attention, learning, memory, and related abilities (4). Notably, research in N Engl J Med and the Lancet suggests that Alzheimer’s disease and dementia may originate in midlife (5, 6). Consequently, early detection, prevention, and treatment of cognitive impairment within the middle-aged population offer a critical opportunity to delay dementia progression (3, 7).

Evidence highlighted by the World Health Organization (WHO) and The Lancet indicates that the development of cognitive impairment and dementia is strongly associated with modifiable risk factors. These encompass unhealthy lifestyle choices, such as physical inactivity, smoking, and excessive alcohol consumption, as well as chronic conditions including hypertension, diabetes, and hyperlipidemia (1, 2). Advances in basic medical research have identified classic biomarkers of cognitive impairment, such as amyloid-*β*, phosphorylated tau, and total tau. Additionally, novel biomarkers including NPTX2 and pro-inflammatory chemokines continue to emerge (8–10). However, the absence of a definitive biomarker for predicting mild cognitive impairment led Pu et al. to develop a nomogram incorporating demographic characteristics, health status, and behavioral data to identify potential predictors (11). Despite these efforts, current detection methods and biomarkers face significant limitations in clinical applicability, underscoring the need for feasible biomarkers to facilitate early intervention.

Cystatin C (CysC), a low-molecular-weight protein produced by nucleated cells, freely traverses the glomerular membrane (12). Since the 1980s, its high sensitivity for detecting impaired renal function (12, 13) has established CysC as a superior marker for estimating glomerular filtration rate (GFR) (14, 15). Meta-analyses by Nair and Yang et al. reported elevated CysC levels in individuals with MCI comorbid with Parkinson’s disease (16, 17). Nevertheless, the relationship between CysC levels and MCI in the natural population remains unclear.

Therefore, this cross-sectional study aims to investigate the association between serum CysC levels and cognitive impairment, and to explore whether this association is independent of renal function (eGFR) and blood pressure, in a natural adult population. Our findings may offer preliminary clues regarding the link between vascular/metabolic factors and cognitive function, which could inform the design of future longitudinal studies aimed at risk prediction.

## 2 Methods

### 2.1 Study design and participants

This study is a cross-sectional analysis. We utilized data from the most recent follow-up in 2023 of the Hanzhong Adolescent Hypertension Study. Details regarding the cohort design have been published previously (18–20). A total of 4,623 Chinese children in 3 towns (Qili, Laojun, and Shayan) were included in this cohort at baseline, which included 26 rural sites in Hanzhong City of Shaanxi province. Participants met these inclusion criteria: no chronic disease history, Mandarin-speaking ability, and voluntary participation. We excluded those with pre-existing chronic conditions or whose parents/guardians declined involvement.

Over 36 years, participants were followed through seven waves: 1989, 1992, 1995, 2005, 2013, 2017, and 2023. While most waves targeted all available participants, the 2005 assessment used systematic sampling (every tenth participant; *n* = 436) as detailed previously (19). Response rates fluctuated across follow-ups: 77.7% (*n* = 3,592) in 1989, 84.8% (*n* = 3,918) in 1992, 82.1% (*n* = 3,794) in 1995, 65.3% (*n* = 3,018) in 2013, 60.1% (*n* = 2,780) in 2017, and 56.7% (*n* = 2,621) in 2023.

The study complies with the Declaration of Helsinki and received ethical approval from the First Affiliated Hospital of Xi’an Jiaotong University (Code: XJTU1AF2015LSL-047). All participants provided written informed consent at each visit (parental/guardian consent for minors). We adhered to STROBE guidelines for observational studies (ClinicalTrials.gov ID: NCT02734472).

**Figure 1:**
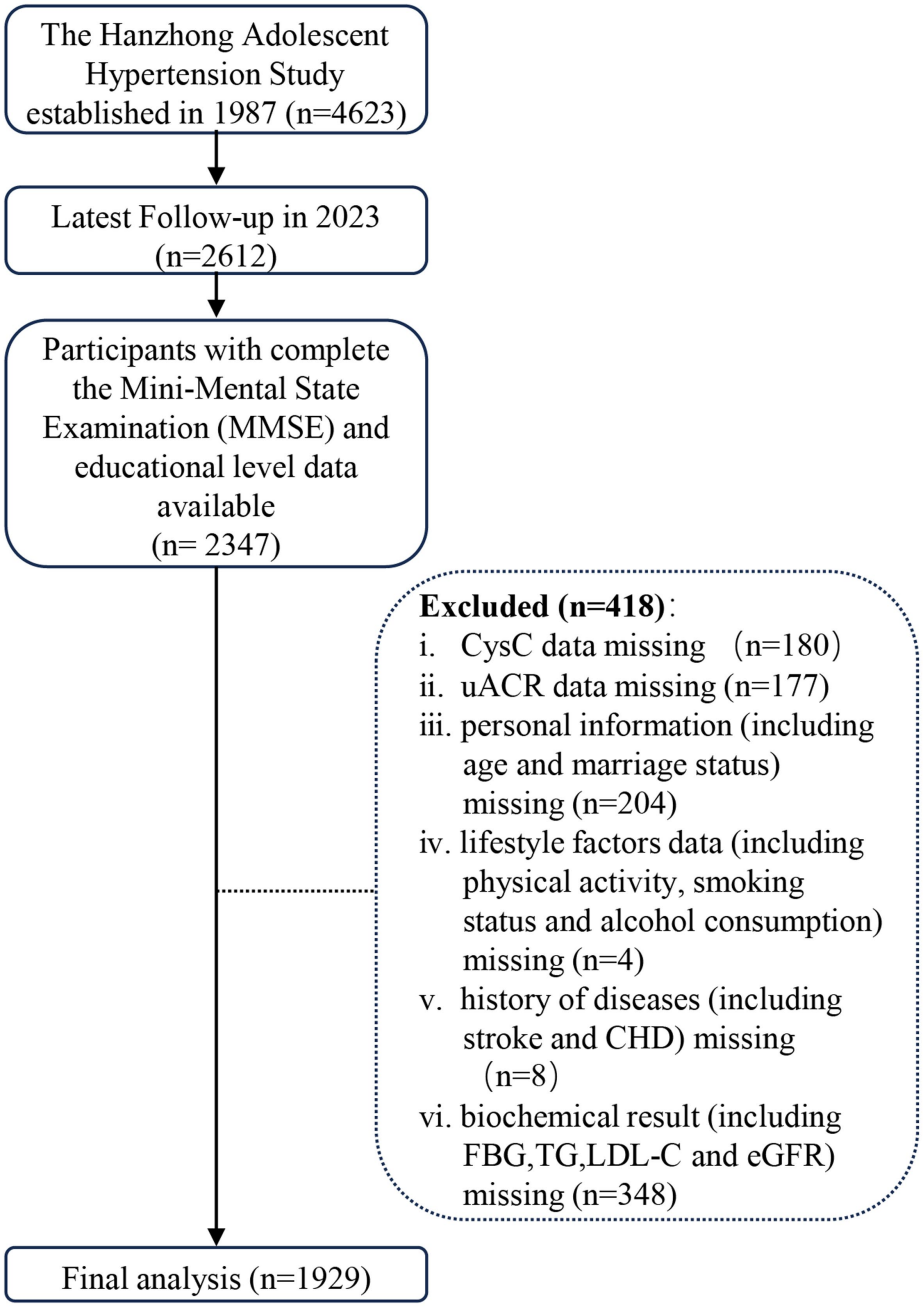
Flowchart for inclusion and exclusion.

Figure 1 illustrates the flow of participant inclusion and exclusion. Given the substantial amount of missing data and concerns that the data were not missing completely at random, we chose to perform a complete-case analysis rather than multiple imputation to avoid introducing bias. Specifically, 2,347 participants completed the Chinese version of the Mini-Mental State Examination (MMSE) and had educational level data available at the 2023 follow-up (Visit 8). We applied exclusions: (1) CysC data missing (*n* = 180); (2) uACR data missing (*n* = 177); (3) personal information (including age and marriage status) missing (*n* = 204); (4) Lifestyle factors data (including physical activity, smoking status and alcohol consumption) missing (*n* = 4); (5) History of diseases [including stroke and CHD (coronary heart disease)] missing (*n* = 8); (6) Biochemical result (including FBG, TG, LDL-C and eGFR) missing (*n* = 348). The final analytical cohort included 1929 participants. Supplementary Table S1 compares the characteristics of excluded and included participants; no substantial differences that would introduce selection bias were observed.

### 2.2 Data collection

This cross-sectional analysis utilized data from the most recent follow-up survey conducted in 2023. The following data were collected: Demographics/lifestyle, including age, sex, education, smoking, alcohol drinking and history of diseases via structured questionnaires; Blood pressure was measured three times in the seated position using mercury sphygmomanometers (19, 21, 22). Height, weight, and waist circumference were measured by trained investigators. Refer to the guidelines for dementia and cognitive impairment in China: the diagnosis and treatment of mild cognitive impairment (23) and previous studies on the Chinese population.

### 2.3 Blood and urine biochemical analyses

We obtained fasting blood and urine samples through peripheral venous puncture and measured biochemical indicators including lipoprotein (a), CysC, fasting blood glucose, total bilirubin, glutamic pyruvic transaminase, glutamic oxaloacetic transaminase, serum creatinine, serum uric acid, total cholesterol, triglyceride, high-density lipoprotein cholesterol (HDL-C), low-density lipoprotein cholesterol (LDL-C), urinary microalbumin and urinary creatinine using the automatic biochemical analyzer (Hitachi, Tokyo, Japan) as described previously (24–26).

### 2.4 Definitions

Refer to the guidelines for dementia and cognitive impairment in China: the diagnosis and treatment of mild cognitive impairment (23) and previous studies on the Chinese population, the cut-off MMSE score points in our research for detecting cognitive impairment are 17 for illiterate individuals, 20 for individuals with 1–6 years of education, and 24 for individuals with 7 or more years of education (27–29). Hypertension was defined as prior diagnosis of hypertension with or without using anti-hypertensives or BP ≥ 140/90 mmHg in 2023 (visit 8). eGFR = 175 × serum creatinine^− 1.234^ × age^− 0.179^ (×0.79 for female). Given the role of CysC in assessing early kidney injury, we stratified participants by albuminuria status (uACR [urinary albumin-to-creatinine ratio] ≥ 30 mg/g) to determine kidney injury status (20, 30, 31).

### 2.5 Covariates

To control for potential confounding effects, the following covariates were included in the multivariable models: (1) Sociodemographic and behavioral characteristics: Gender, age, marital status, physical activities, smoking habits, alcohol consumption; (2) Anthropometric parameters: BMI, mean BMI, mean SBP; (3) Clinical history: stroke, coronary heart disease (CHD); (4) Biochemical assays: fasting blood glucose (FBG), triglyceride (TG), LDL-C and eGFR.

### 2.6 Statistical analyses

Median with interquartile ranges (IQR) was used to describe the continuous variables, and the Mann–Whitney U-test was used to assess the group differences. Frequency and percentage were used to describe the categorical variables, and the chi-square test assessed group differences.

We used multiple logistic regression to examine the association between CysC levels and cognitive impairment as odds ratios (ORs) with corresponding 95% confidence intervals (CIs). The models followed a sequential adjustment strategy: (1) Model 1 was unadjusted; (2) Model 2 was adjusted for basic demographic and clinical factors, including age, gender, mean SBP, and mean BMI; (3) Model 3 further extended the adjustment by incorporating the covariates from Model 2 plus additional variables pertaining to lifestyle, cardiovascular history, and extended biochemical profiles, specifically: marital status, physical activities, smoking habits, alcohol consumption, stroke, CHD, FBG, TG, LDL-C, and eGFR.

We calculated Pearson correlation coefficients between CysC and all covariates (Supplementary Table S2). The variance inflation factor (VIF) was computed to assess multicollinearity; values below 10 indicate the absence of severe multicollinearity. The correlation between CysC and eGFR was −0.288, with corresponding VIF values of 1.13 for CysC and 1.15 for eGFR.

To further explore the heterogeneity in the association between CysC and cognitive impairment, we conducted subgroup and interaction analyses. CysC and albuminuria serve as biomarkers for detecting early renal impairment in individuals with preserved or mildly reduced eGFR (20, 30, 31). This study cohort comprised a natural population sample, with a limited number of participants with eGFR < 90 mL/min/1.73 m^2^. Consequently, the association between serum CysC levels and cognitive impairment was examined, stratified by albuminuria status (uACR < 30 mg/g vs. ≥ 30 mg/g). In the interaction analysis, the likelihood ratio test was used to assess the significance of interaction effects. Furthermore, to account for the potential confounding effects of gender, age, alcohol consumption, and chronic conditions on cognitive function, subgroup analyses were stratified by these variables. The potential nonlinear relationship between CysC and cognitive impairment was evaluated using restricted cubic splines (RCS) with 3 knots placed at the 10th, 50th, and 90th percentiles. In both the subgroup and RCS analyses, all covariates included in Model 3 were adjusted for as potential confounders, except for the stratification variable itself in the subgroup analyses. To enhance the robustness of the findings, we platformed the sensitivity analysis after excluding the participants receiving pharmacological treatment for hypertension, diabetes, or dyslipidemia. Data processing and analysis were performed using R (version 4.5.1) or Stata (version 17.0), *p*-values < 0.05 were considered statistically significant.

## 3 Results

### 3.1 The characteristics of the participants grouped according to cognitive impairment

A total of 1,929 participants were included in this study, of whom 1,077 (45.89%) were female. The median age was 49 (32–37) years. The median serum CysC level was 1.03 (0.88–1.19) mg/L. Based on its distribution, CysC was categorized into quartiles: Q1 (<0.88 mg/L), Q2 (0.88 to <1.03 mg/L), Q3 (1.03 to <1.19 mg/L), and Q4 (≥1.19 mg/L). CysC levels were also standardized (converted to z-scores) for some analyses. Cognitive impairment was identified in 149 participants (7.72%).

Table 1 summarizes demographic, clinical, and laboratory characteristics of participants with and without cognitive impairment. Compared to cognitively normal participants, those with cognitive impairment were significantly older. They also demonstrated higher mean SBP and mean BMI over the 36-year follow-up period. Furthermore, participants with cognitive impairment exhibited significantly higher levels of Lp(a) and CysC. Lower urinary creatinine levels and significantly lower MMSE scores were also observed in the cognitive impairment group.

**Table 1:** Characteristics of participants grouped by cognitive impairment.

Variable	Total (*n* = 1929)	Without cognitive impairment (*n* = 1780)	With cognitive impairment (*n* = 149)	*p*-value
Age, years	49 (45–50)	48 (45–50)	50 (48–51)	**<0.001**
SBP, mmHg	125 (114.5–136.5)	125 (114–136.5)	127.5 (117–140)	**0.046**
DBP, mmHg	83 (75–91)	83 (75–91)	82.5 (77–91)	0.481
HR, beats/min	77 (70.33–85)	77 (70.67–85.33)	77 (69.5–84)	0.856
MeanSBP, mmHg	116.43 (109.9–124.09)	116.28 (109.79–123.79)	119.62 (110.84–128.68)	**0.006**
Mean BMI, kg/m^2^	21.38 (19.8–22.86)	21.36 (19.77–22.81)	21.85 (20.25–23.6)	**0.011**
CysC, mg/L	1.03 (0.88–1.19)	1.03 (0.88–1.19)	1.1 (0.92–1.29)	**<0.001**
Fasting insulin, mU/L	14.67 (11.75–18.82)	14.65 (11.65–18.83)	15.25 (12.16–18.66)	0.600
hs-CRP, mg/L	0.68 (0.33–1.49)	0.68 (0.33–1.48)	0.68 (0.34–1.69)	0.718
FBG, mmol/L	5.31 (4.95–5.78)	5.31 (4.95–5.78)	5.36 (5.03–5.94)	0.194
Serum Cre, μmol/L	73.3 (64.6–82.1)	73.4 (64.6–82.03)	71.9 (63.3–82.2)	0.705
Serum UA, μmol/L	282 (232.5–337.6)	282.6 (232.07–338.3)	279.5 (236.5–335.2)	0.975
TC, mmol/L	4.73 (4.24–5.3)	4.73 (4.24–5.29)	4.77 (4.21–5.38)	0.542
TG, mmol/L	1.57 (1.12–2.25)	1.57 (1.13–2.26)	1.48 (1.08–2.16)	0.269
HDL, mmol/L	1.13 (0.98–1.31)	1.13 (0.98–1.31)	1.14 (0.98–1.36)	0.656
LDL, mmol/L	2.63 (2.23–3.05)	2.62 (2.23–3.05)	2.69 (2.27–3.08)	0.603
Lp(a), mg/L	160 (91–272)	159 (90–266.25)	182 (112–318)	**0.020**
MMSE score	28 (26–29)	28 (27–29)	24 (22–24)	**<0.001**
eGFR, mL/min/1.73m^2^	99.5 (90.9–110)	99.5 (91.07–110)	99 (89.6–110)	0.754
uACR, mg/g	4.66 (1.79–14.02)	4.6 (1.81–13.71)	6.65 (1.74–20.95)	0.181
Gender				0.864
Female	891 (46.19%)	821 (46.12%)	70 (46.98%)	
Male	1,038 (53.81%)	959 (53.88%)	79 (53.02%)	
Smoking, *n* (%)				0.931
No	1,162 (60.24%)	1,073 (60.28%)	89 (59.73%)	
Yes	767 (39.76%)	707 (39.72%)	60 (40.27%)	
Drinking, *n* (%)				**0.033**
No	1,423 (73.77%)	1,302 (73.15%)	121 (81.21%)	
Yes	506 (26.23%)	478 (26.85%)	28 (18.79%)	
Married status, *n* (%)				0.087
Unmarried	13 (0.67%)	11 (0.62%)	2 (1.34%)	
Married	1839 (95.33%)	1,695 (95.22%)	144 (96.64%)	
Divorced	62 (3.21%)	61 (3.43%)	1 (0.67%)	
Widowed	15 (0.78%)	13 (0.73%)	2 (1.34%)	
Physical activities, *n* (%)				**0.008**
No activity	237 (12.29%)	224 (12.58%)	13 (8.72%)	
Mild	799 (41.42%)	751 (42.19%)	48 (32.21%)	
Moderate	675 (34.99%)	614 (34.49%)	61 (40.94%)	
Vigorous	218 (11.30%)	191 (10.73%)	27 (18.12%)	
Education, *n* (%)				**<0.001**
Illiteracy	1 (0.05%)	1 (0.06%)	0 (0.00%)	
Primary school	142 (7.36%)	133 (7.47%)	9 (6.04%)	
Junior high school	1,158 (60.03%)	1,043 (58.60%)	115 (77.18%)	
High/secondary school	413 (21.41%)	392 (22.02%)	21 (14.09%)	
Post-secondary education	213 (11.04%)	209 (11.74%)	4 (2.68%)	
Master’s degree or higher	2 (0.10%)	2 (0.11%)	0 (0.00%)	
Stoke, *n* (%)				0.664
No	1909 (98.96%)	1762 (98.99%)	147 (98.66%)	
Yes	20 (1.04%)	18 (1.01%)	2 (1.34%)	
CHD, *n* (%)				1.000
No	1924 (99.74%)	1775 (99.72%)	149 (100.00%)	
Yes	5 (0.26%)	5 (0.28%)	0 (0.00%)	
Hypertension, *n* (%)				0.108
No	1,248 (64.70%)	1,161 (65.22%)	87 (58.39%)	
Yes	681 (35.30%)	619 (34.78%)	62 (41.61%)	
Diabetes, *n* (%)				0.880
No	1763 (91.39%)	1,627 (91.40%)	136 (91.28%)	
Yes	166 (8.61%)	153 (8.60%)	13 (8.72%)	
Hyperlipidemia, *n* (%)				0.551
No	960 (49.77%)	882 (49.55%)	78 (52.35%)	
Yes	969 (50.23%)	898 (50.45%)	71 (47.65%)	
Drug treatment, *n* (%)				0.334
No	1,646 (85.33%)	1,523 (85.56%)	123 (82.55%)	
Yes	283 (14.67%)	257 (14.44%)	26 (17.45%)	
Albuminuria, *n* (%)				0.104
No	1,668 (86.47%)	1,546 (86.85%)	122 (81.88%)	
Yes	261 (13.53%)	234 (13.15%)	27 (18.12%)	

HR, heart rate; SBP, systolic blood pressure; DBP, diastolic blood pressure; Mean SBP, mean SBP of 36 years; BMI, body mass index; MMSE, Mini-Mental State Examination; CysC, cystatin C; hs-CRP, high-sensitivity C-reactive protein; FBG, fasting blood glucose; Cre, Creatinine; UA, uric acid; TC, total cholesterol; TG, triglycerides; HDL-C, high-density lipoprotein cholesterol; LDL-C, low-density lipoprotein cholesterol; eGFR, estimated glomerular filtration rate; uACR, urinary microalbumin-to-creatinine ratio. Bold *p*-values indicate statistically significant differences.

### 3.2 Association between CysC levels and cognitive impairment

Table 2 presents the association between midlife CysC levels and cognitive impairment. In the unadjusted model, each SD increase in CysC level was significantly associated with greater odds of cognitive impairment (OR = 1.43, 95% CI: 1.22–1.68; *p* < 0.001). This association persisted in the full adjustment model (adjusted OR = 1.49, 95% CI: 1.24–1.79; *p* < 0.001). Participants in the highest CysC quartile had 1.79-fold greater odds of cognitive impairment compared to the lowest quartile (adjusted OR = 1.79 95% CI: 1.09–2.98; *p* = 0.022). Complete specifications of the multiple regression models, including model equations, are provided in Supplementary Table S3. Supplementary Table S4 shows the association between CysC and cognitive impairment without adjustment for eGFR.

**Table 2:** ORs and 95% CIs of cognitive impairment.

Variable	Model 1			Model 2			Model 3		
	OR	95% CI	*p*-value	OR	95% CI	*p*-value	OR	95% CI	*p*-value
CysC, mg/L	3.40	1.95–5.95	**<0.001**	3.15	1.80–5.51	**<0.001**	3.90	2.12–7.31	**<0.001**
CysC (z-score)	1.43	1.22–1.68	**<0.001**	1.40	1.19–1.64	**<0.001**	1.49	1.24–1.79	**<0.001**
CysC quartiles									
Q1	1.00 (Ref)	–	–	1.00 (Ref)	–	–	1.00 (Ref)	–	–
Q2	0.76	0.44–1.31	0.332	0.76	0.44–1.32	0.334	0.78	0.44–1.35	0.368
Q3	1.37	0.85–2.22	0.201	1.34	0.83–2.19	0.232	1.45	0.89–2.39	0.142
Q4	1.67	1.06–2.68	**0.028**	1.56	0.98–2.50	0.062	1.79	1.09–2.98	**0.022**

CysC, cystatin C; OR, Odds ratio; CI, confidence interval. Model 1: univarate. Model 2: adjusted for Gender, age, mean BMI, mean SBP. Model 3: Model 2 + marital status, physical activities, smoking habits, alcohol consumption, stroke, CHD, fasting blood glucose, triglyceride, LDL-C and eGFR. Bold *p*-values indicate statistically significant differences.

### 3.3 Multivariate adjusted ORs for the association between CysC levels and cognitive impairment in subgroups with and without albuminuria

Subgroup analyses were performed to examine the association between serum CysC and cognitive impairment across albuminuria-defined subgroups. Participants were categorized into albuminuria (*n* = 261) and non-albuminuria (*n* = 1,668) subgroups. Baseline characteristics of participants stratified by albuminuria status are shown in Supplementary Table S5. Cognitive impairment prevalence was 10.34% (27/261) in the albuminuria group and 7.31% (122/1668) in the non-albuminuria group. Participants with albuminuria exhibited a higher burden of comorbidities, including hypertension, diabetes, and dyslipidemia, along with elevated long-term systolic blood pressure (SBP), body mass index (BMI), fasting blood glucose (FBG), high-sensitivity C-reactive protein (hs-CRP), serum creatinine, and triglyceride levels, but lower HDL-C levels.

Results of the subgroup and interaction analyses by albuminuria status are presented in Table 3. In the non-albuminuria subgroup, each SD increase in CysC was significantly associated with greater odds of cognitive impairment after full adjustment (OR = 1.48, 95% CI: 1.22–1.80; *p* < 0.001). Participants in the highest CysC quartile had 1.75-fold greater odds of cognitive impairment compared with the lowest quartile (OR = 1.75, 95% CI: 1.03–3.03; *p* = 0.040). In the albuminuria subgroup, no significant associations between CysC and cognitive impairment were observed in any model. In the interaction analysis, the associations of both CysC levels and its z-score with cognitive impairment differed significantly according to albuminuria status (*p* for interaction = 0.028 for each). However, when CysC was analyzed in quartiles (Q1-Q4), albuminuria status did not significantly modify the association with cognitive impairment (*p* for interaction = 0.074).

**Table 3:** ORs and 95% CIs of cognitive impairment in non-albuminuria subgroup and albuminuria subgroup.

Variable	Event/N	Model 1			Model 2			Model 3		
		OR	95% CI	*p*-value	OR	95% CI	*p*-value	OR	95% CI	*p*-value
Non-albuminuria subgroup										
CysC, mg/L	122/1668 (7.23%)	3.83	2.07–7.24	**<0.001**	3.47	1.86–6.55	**<0.001**	3.86	2.01–7.58	**<0.001**
CysC (z-score)		1.48	1.24–1.78	**<0.001**	1.44	1.20–1.73	**<0.001**	1.48	1.22–1.80	**<0.001**
CysC quartiles										
Q1		1.000 (Ref)	–	–	1.000 (Ref)	–	–	1.000 (Ref)	–	–
Q2		1.03	0.58–1.82	0.923	0.98	0.55–1.75	0.946	0.94	0.52–1.69	0.828
Q3		1.45	0.86–2.47	0.170	1.32	0.78–2.29	0.306	1.35	0.79–2.35	0.278
Q4		1.77	1.06–2.99	**0.030**	1.63	0.97–2.78	0.069	1.75	1.03–3.03	**0.040**
Albuminuria subgroup										
CysC, mg/L	27/261 (8.43%)	1.27	0.48–2.58	0.545	1.26	0.47–2.62	0.564	0.99	0.34–2.18	0.981
CysC (z-score)		1.07	0.81–1.32	0.545	1.07	0.80–1.32	0.564	1.00	0.73–1.25	0.981
CysC quartiles										
Q1		1.000 (Ref)	–	–	1.000 (Ref)	–	–	1.000 (Ref)	–	–
Q2		0.15	0.01–0.86	0.077	0.14	0.01–0.84	0.072	0.13	0.01–0.78	0.061
Q3		1.59	0.54–4.80	0.402	1.83	0.59–5.81	0.293	2.29	0.68–8.13	0.184
Q4		1.34	0.50–3.81	0.571	1.27	0.46–3.72	0.652	1.33	0.42–4.39	0.626
*p* for interaction of CysC		**0.027**			**0.046**			**0.028**		
*p* for interaction of CysC (z_score)		**0.027**			**0.046**			**0.028**		
*p* for interaction of CysC quartiles		0.137			0.107			0.074		

CysC, cystatin C; OR, Odds ratio; CI, confidence interval. Model 1: univarate. Model 2: adjusted for Gender, age, mean BMI, mean SBP. Model 3: Model 2 + marital status, physical activities, smoking habits, alcohol consumption, stroke, CHD, fasting blood glucose, triglyceride, LDL-C and eGFR. Bold *p*-values indicate statistically significant differences.

**Figure 2:**
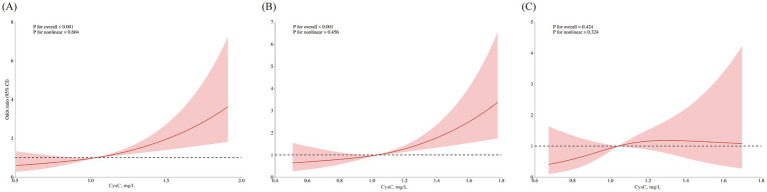
Nonlinear association of serum CysC with cognitive impairment across subgroups. Restricted cubic spline plots showing the association of serum cystatin C with the odds (log-scale) of cognitive impairment in a cross-sectional analysis. All models are adjusted for gender, age, mean BMI, mean SBP, marital status, physical activities, smoking habits, alcohol consumption, stroke, CHD, fasting blood glucose, triglyceride, LDL-C, and eGFR. **(A)** Total population. **(B)** Non-albuminuria subgroup. **(C)** Albuminuria subgroup.

The patterns of association derived from restricted cubic spline analyses are illustrated in Figure 2. A near-linear increasing trend with tight confidence bands was observed in all participants and the non-albuminuria subgroup, aligning with their significant linear *p*-values (both <0.001) and non-significant nonlinearity tests (*p* = 0.604 and 0.456). Conversely, the albuminuria subgroup displayed a potential non-linear (rising then falling) trend with broad confidence intervals. Statistically, neither linear nor nonlinear associations were significant in this subgroup (*p* for linearity >0.05, *p* for nonlinearity = 0.324). The precision of the estimate in this group was lower, as reflected by the wide CIs.

### 3.4 Sensitivity analysis and subgroup analysis

Considering the potential confounding effects of antihypertensive, hypoglycemic, or lipid-lowering medications on renal and cognitive function, we excluded 283 participants with medication using (Supplementary Table S6). The association between CysC and cognitive impairment remained significant after the exclusion. In fully adjusted models, each SD increase in CysC was significantly associated with higher cognitive impairment risk (OR = 1.45, 95% CI: 1.18–1.81; *p* < 0.001). Participants in the highest CysC quartile exhibited 2.0-fold greater odds of impairment compared to the lowest quartile (OR = 2.01, 95% CI: 1.10–3.75; *p* = 0.024; Supplementary Table S7).

In the subgroup analyses, all covariates, except for the stratification variable itself, were adjusted for as potential confounders. Baseline characteristics of participants stratified by albuminuria status, sex, alcohol consumption, and hypertension status are presented in Supplementary Tables S8–S10, respectively. The associations of both CysC levels and its z-score with cognitive impairment are presented in Supplementary Figure S1. Subgroup analyses were performed according to sex, alcohol consumption status, and hypertension status. A per-SD increase in the CysC z-score remained significantly associated with greater odds of cognitive impairment across all prespecified subgroups: among men (adjusted OR = 1.58, 95% CI: 1.24–2.03) and women (OR = 1.34, 95% CI: 1.04–1.73); among alcohol consumers (OR = 2.23, 95% CI: 1.33–3.75) and non-consumers (OR = 1.41, 95% CI: 1.16–1.71); and among participants with hypertension (OR = 1.77, 95% CI: 1.31–2.39) and those without (OR = 1.39, 95% CI: 1.11–1.73; all *p*-values < 0.005). The interaction terms for these subgroup variables were not statistically significant (all *p* for interaction > 0.05), indicating that these factors did not significantly modify the main association.

## 4 Discussion

Our study demonstrates the association between CysC levels and cognitive impairment assessed by MMSE after comprehensive covariate adjustment in the midlife natural population from Hanzhong Adolescent Hypertension Cohort: Compared to the lowest CysC quartile, participants in the highest quartile showed a noteworthy elevation in the prevalence of cognitive impairment, and the association exhibits in participants without albuminuria in subgroup analysis.

The CysC-cognition association was substantially amplified in the hypertension population compared to normotensive individuals. It is in line with the previous conclusion that elevated blood pressure increases the risk of cognitive impairment, especially for population with hypertension and without renal damage (38–41). Furthermore, our analyses revealed amplified associations between elevated CysC levels and cognitive impairment in male participants and alcohol consumers. This underscores alcohol’s underrecognized role as a potential modifier of cognitive pathology (39, 42). A significant interaction was observed between CysC levels and albuminuria status, revealing that the association was more pronounced in individuals without albuminuria. Several non-mutually exclusive explanations may account for this observation. First, the albuminuria subgroup had a smaller sample size and fewer cognitive impairment events, resulting in wider confidence intervals and reduced statistical power to detect a significant association. Second, although we adjusted for eGFR, residual confounding from unmeasured factors, such as the severity of co-existing chronic conditions or systemic inflammation levels, may have obscured the relationship within the albuminuria group. Third, distinct biological mechanisms may operate at different stages of renal and vascular disease. In individuals without albuminuria, elevated CysC may primarily reflect early subclinical vascular injury or neuroinflammation, serving as a sensitive early marker for cognitive risk. In contrast, in individuals with established albuminuria—a marker of more advanced renal damage—cognitive impairment may be driven predominantly by stronger competing risk factors, such as the accumulation of uremic toxins or severe cardiovascular complications, thereby attenuating the measurable independent contribution of CysC.

Pacholko et al. (43–45) established elevated blood pressure as a well-documented risk factor for cognitive impairment, a conclusion consistent with our findings. The kidneys represent prime target organs for hypertension-induced damage (46–48), with albuminuria serving as a key biomarker of renal impairment (49, 50). These findings in subgroup analysis carry important clinical implications. For non-albuminuric patients, CysC monitoring may offer earlier detection of neuroinflammatory processes preceding cognitive decline. Our finding of a strong association between elevated CysC and cognitive impairment in the non-albuminuric subgroup suggests that, in populations with hypertension but without advanced renal damage, CysC may be a relevant biomarker of concurrent cerebrovascular burden. Future prospective studies are needed to determine if monitoring CysC levels over time can help identify individuals at a higher risk of progressive cognitive impairment.

In the early stages of hypertension, the condition is characterized by slightly elevated blood pressure and a short duration of illness. During this period, there is typically without renal damage. We hope that the conclusion of our research can raise more attention to cognitive impairment at this stage. A multicenter study pointed that exposure to higher BP levels from young to midlife is associated with worse cognitive function in midlife (32). Our study extends this paradigm by demonstrating that sustained BP elevation from adolescence through midlife exerts particularly detrimental effects. While the precise pathological mechanisms linking hypertension and cognitive decline remain incompletely characterized (33), we hypothesize a potential cerebrovascular pathway: chronic elevation in blood pressure may contribute to cerebral small vessel disease and blood–brain barrier dysfunction, processes in which CysC may serve as a biomarker. This hypothesis, if confirmed by longitudinal studies, would have two important implications: First, it would suggest that long-term BP trajectories might be more informative than single measurements in understanding the development of cognitive impairment. Second, it would underscore the potential importance of early-life cardiovascular health management for preserving cognitive function later in life. Future research with repeated measures is needed to directly test these possibilities.

The MMSE scale remains a classic screening tool for cognitive impairment. However, defining cognitive impairment solely by MMSE scores may increase false-positive identifications, potentially leading to inaccurate targeting of preventive interventions for dementia. To enhance diagnostic precision, our study defined cognitive impairment by integrating MMSE scores with educational attainment levels. This combined approach demonstrates improved accuracy and feasibility for identifying cognitive impairment in middle-aged and elderly Chinese populations with significant educational disparities—a methodology meriting broader implementation in other underdeveloped regions. However, a key limitation of our study is its reliance on the MMSE for defining cognitive impairment. The MMSE has well-recognized limitations, including ceiling effects in highly educated individuals and insensitivity to early or domain-specific cognitive decline, particularly in executive function and attention. This relative insensitivity likely results in non-differential misclassification, where some participants with genuine mild cognitive impairment are incorrectly classified as cognitively normal. Such misclassification typically biases the observed associations toward the null (attenuation), meaning that the true strength of the association between CysC and cognitive impairment may be stronger than what we reported. Other instruments, such as the Montreal Cognitive Assessment (MoCA), offer higher sensitivity for mild cognitive impairment, and the Digit Symbol Substitution Test (DSST) provides a more comprehensive assessment of attention and processing speed. However, given the large scale of our cohort, the participants’ generally low and heterogeneous educational backgrounds, and the practical constraints of field work in an underdeveloped region, the MMSE was chosen for its superior feasibility in large-scale population screening for cognitive impairment and dementia.

The relationship between CysC and cognitive function remains inconclusive (34). Contrary to our findings, GAUTHIER et al. proposed that CysC exerts protective effects against neurodegeneration, learning deficits, and cognitive impairment (34–36), with lower serum CysC levels associated with increased disease risk (37). However, the data in these prior studies are primarily derived from hospital-based patient cohorts, potentially introducing selection bias and confounding effects from underlying comorbidities on both CysC levels and cognitive function (51–53). Previous studies have demonstrated that elderly individuals aged 70–79 years with elevated CysC levels exhibited poorer MMSE and DSST scores, showing greater cognitive impairment over 7-year follow-up compared to those with lower CysC levels. BANG and GREGORY et al. suggested that cognitive impairment may originate in midlife, a view supported by the Lancet Commission’s recommendation to initiate dementia prevention during midlife (2). Our study focused on a middle-aged natural population with lower cognitive impairment prevalence. By combining MMSE scores with educational attainment, we established a methodology that enables more accurate and efficient identification of cognitive impairment in questionnaire-based screenings. Notably, the association between elevated CysC levels and cognitive impairment remained significant and was even stronger after stratification by albuminuria status, blood pressure, age, sex, and lifestyle factors. These subgroup analyses indicate that the association between CysC and cognitive impairment warrants renewed attention and broader clinical application.

American Academy of Neurology (AAN) (54–56) and the Lancet Commission (2) have reported insufficient evidence to recommend pharmacological interventions for mild cognitive impairment, with no proven cognition-enhancing effects from specific treatments. Blood-based biomarkers offer broader acceptability and applicability for detection (2), presenting potential advantages in cognitive impairment screening. As CysC is routinely screened in natural populations, we propose its elevation as an early warning biomarker for cognitive impairment risk. This approach demonstrates operational feasibility with minimal added public health burden. We recommend healthcare providers: Firstly, screen high-risk populations through CysC monitoring; Secondly, identify cognitive impairment using MMSE scores adjusted for educational attainment; Thirdly, guide at-risk individuals to modify risk factors (e.g., obesity, hypertension, alcohol overconsumption, air pollutants (2)). This integrated strategy may enable earlier detection of dementia and Alzheimer’s disease, thereby reducing the public health burden.

Our study has several limitations. First, as a single-center investigation, a larger sample size is required to refine the analytical models. Further research is needed to establish the critical threshold for CysC levels to enable precise identification of high-risk cognitive impairment populations. Second, cognitive impairment was defined using MMSE scores adjusted for educational attainment. This approach lacks the diagnostic specificity of specialized clinical assessments such as neuroimaging and electrophysiological examinations. Third, although this cohort was established in 1987, cognitive function assessments were primarily concentrated at the most recent follow-up in 2023. Extended longitudinal follow-up is necessary to elucidate the long-term impact of elevated CysC on cognitive trajectories. Finally, the absence of mechanistic studies limits biological validation. Based on established biochemical functions of CysC and existing evidence, we hypothesize its involvement in amyloid-*β* metabolism (57–59) and neuronal degeneration processes (60). However, this mechanistic explanation remains speculative and must be considered a hypothesis requiring validation through well-designed basic science experiments or dedicated long-term longitudinal studies.

## 5 Conclusion

In conclusion, our research found that Elevated CysC is associated with cognitive impairment assessed by MMSE in this midlife natural population cohort, and this association is exhibited stronger in participants without albuminuria. Furthermore, our subgroup analyses further highlight the heterogeneity of this association across different populations. Collectively, these findings underscore the potential importance of enhancing cognitive function screening among individuals with elevated CysC levels, with the aim of early identification of those at risk for Alzheimer’s disease and dementia.

## Data Availability

The raw data supporting the conclusions of this article will be made available by the authors, without undue reservation.
