# Supplementary material for: Serum cystatin C and cognitive function in midlife: results from the Hanzhong Adolescent Hypertension Study
Source: Front Neurol. 2026 Feb 10;17:1739512. doi: 10.3389/fneur.2026.1739512 (PMC12929118; doi:10.3389/fneur.2026.1739512)
Supplement: Supplementary file 1 [file Table_1.docx]

**Supplemental Material**

**Supplemental Figure S1.** Subgroup analysis using multivariable logistic regression examined the association between CysC and cognitive impairment.

**Table S1** Characteristics of included and excluded participants

**Table S2** Pearsson correlation coefficient between CysC and covariates

**Table S3** Association of CysC, covariate and cognitive impairment

**Table S4** Association of CysC and cognitive impairment excluded the covariate of eGFR

**Table S5** Characteristics of participants grouped by albuminuria.

**Table S6** Characteristics of participants grouped by drug treatment.

**Table S7** ORs and 95% CIs of cognitive impairment without durg treatment.

**Supplemental Table S8.** Characteristics of participants grouped by gender.

**Supplemental Table S9.** Characteristics of participants grouped by alcohol consumption.

**Supplemental Table S10.** Characteristics of participants grouped by hypertension.

**Figure S1.** Association of Serum Cystatin C with Cognitive Impairment Across Prespecified Subgroups.


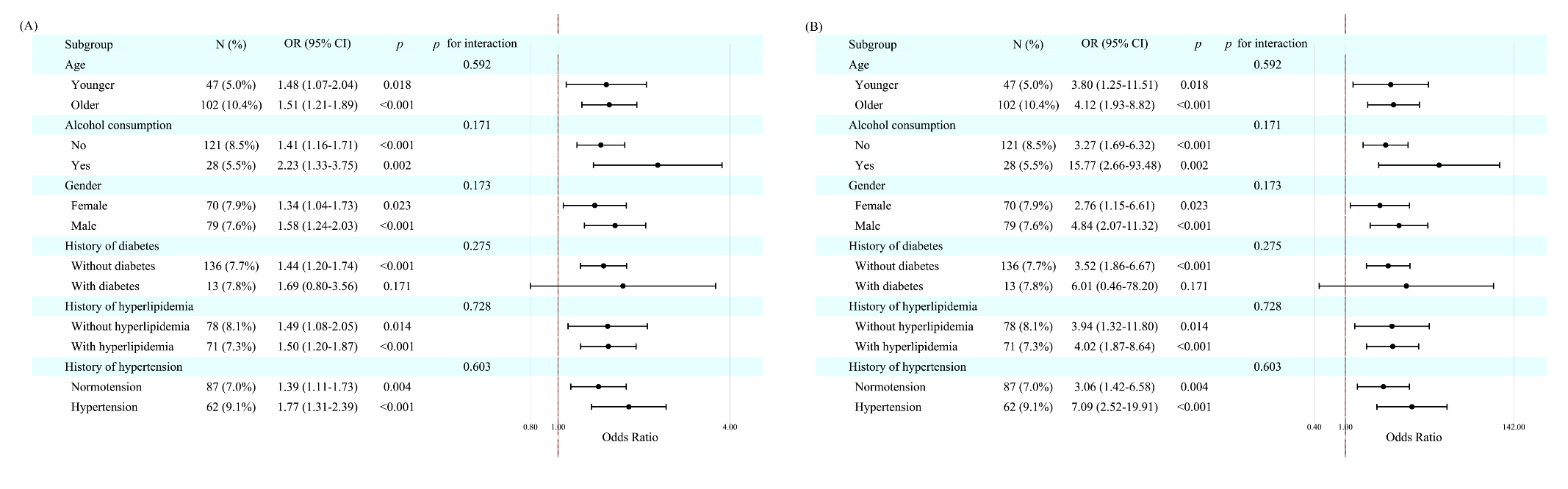


(A) Forest plot of adjusted odds ratios (ORs) for cognitive impairment per 1-standard deviation increase in the serum cystatin C z-score. (B) Forest plot of adjusted ORs for cognitive impairment per 1-mg/L increase in the absolute serum cystatin C concentration. All models were adjusted for age, gender, mean SBP, and mean BMI, marital status, physical activities, smoking habits, alcohol consumption, stroke, CHD, FBG, TG, LDL-C, and eGFR—except for the stratification variable itself in each subgroup analysis. CI, confidence interval; OR, odds ratio.

**Table S1** Characteristics of included and excluded participants

| Variable | Completed the MMSE (n=2347) | Excluded (n=418) | Final analytical sample (n=1929) | *p*-value |
| --- | --- | --- | --- | --- |
| Age, years | 49 (45-50) | 48 (45-51) | 49 (45-50) | 0.903 |
| SBP, mmHg | 125 (114-137) | 123.5 (113.5-137.5) | 125 (114.5-136.5) | 0.682 |
| DBP, mmHg | 83 (75-91) | 82.5 (75-92) | 83 (75-91) | 0.893 |
| HR, beats/min | 77.33 (70.67-85.33) | 79.33 (71-86.08) | 77 (70.33-85) | **0.035** |
| MeanSBP, mmHg | 116.44 (109.82-124.38) | 116.44 (109.7-125.62) | 116.43 (109.9-124.09) | 0.752 |
| Mean BMI, kg/m² | 21.38 (19.77-22.92) | 21.31 (19.72-23.17) | 21.38 (19.8-22.86) | 0.873 |
| CysC, mg/L | 1.03 (0.88-1.19) | 1.02 (0.89-1.19) | 1.03 (0.88-1.19) | 0.508 |
| Fasting insulin, mU/L | 14.64 (11.69-18.75) | 14.45 (11.37-18.57) | 14.67 (11.75-18.82) | 0.729 |
| hs-CRP, mg/L | 0.69 (0.33-1.5) | 0.8 (0.38-1.55) | 0.68 (0.33-1.49) | 0.323 |
| FBG, mmol/L | 5.3 (4.94-5.79) | 5.26 (4.88-5.87) | 5.31 (4.95-5.78) | 0.202 |
| Serum Cre, μmol/L | 73.4 (64.8-82.2) | 73.6 (66.77-83.03) | 73.3 (64.6-82.1) | 0.146 |
| Serum UA, μmol/L | 282.4 (232.4-339.6) | 290.8 (231.75-349.67) | 282 (232.5-337.6) | 0.191 |
| TC, mmol/L | 4.74 (4.24-5.29) | 4.77 (4.27-5.27) | 4.73 (4.24-5.3) | 0.951 |
| TG, mmol/L | 1.56 (1.11-2.24) | 1.53 (1.05-2.18) | 1.57 (1.12-2.25) | 0.666 |
| HDL, mmol/L | 1.13 (0.98-1.31) | 1.12 (0.97-1.32) | 1.13 (0.98-1.31) | 0.843 |
| LDL, mmol/L | 2.64 (2.24-3.06) | 2.67 (2.26-3.08) | 2.63 (2.23-3.05) | 0.744 |
| Lp(a), mg/L | 157 (90-267) | 135 (86-237.25) | 160 (91-272) | **0.021** |
| MMSE score | 28 (26-29) | 28 (26-29) | 28 (26-29) | 0.602 |
| eGFR, mL/min/1.73m² | 99.45 (90.97-110) | 99 (91.75-108) | 99.5 (90.9-110) | 0.889 |
| uACR, mg/g | 4.6 (1.76-13.85) | 4.08 (1.57-12.22) | 4.66 (1.79-14.02) | 0.180 |
| Gender |  |  |  | 0.552 |
| Female | 1077 (45.89%) | 186 (44.50%) | 891 (46.19%) |  |
| Male | 1270 (54.11%) | 232 (55.50%) | 1038 (53.81%) |  |
| Smoking, n (%) |  |  |  | 0.138 |
| No | 1397 (59.52%) | 235 (56.22%) | 1162 (60.24%) |  |
| Yes | 950 (40.48%) | 183 (43.78%) | 767 (39.76%) |  |
| Drinking, n (%) |  |  |  | 0.077 |
| No | 1712 (72.94%) | 289 (69.14%) | 1423 (73.77%) |  |
| Yes | 633 (26.97%) | 127 (30.38%) | 506 (26.23%) |  |
| Married status, n (%) |  |  |  | 0.069 |
| Unmarried | 21 (0.89%) | 8 (1.91%) | 13 (0.67%) |  |
| Married | 2220 (94.59%) | 381 (91.15%) | 1839 (95.33%) |  |
| Divorced | 78 (3.32%) | 16 (3.83%) | 62 (3.21%) |  |
| Widowed | 19 (0.81%) | 4 (0.96%) | 15 (0.78%) |  |
| Physical activities, n (%) |  |  |  | 0.802 |
| No activity | 289 (12.31%) | 52 (12.44%) | 237 (12.29%) |  |
| Mild | 981 (41.80%) | 182 (43.54%) | 799 (41.42%) |  |
| Moderate | 815 (34.73%) | 140 (33.49%) | 675 (34.99%) |  |
| Vigorous | 260 (11.08%) | 42 (10.05%) | 218 (11.30%) |  |
| Education, n (%) |  |  |  | 0.467 |
| Illiteracy | 2 (0.09%) | 1 (0.24%) | 1 (0.05%) |  |
| Primary school | 181 (7.71%) | 39 (9.33%) | 142 (7.36%) |  |
| Junior high school | 1405 (59.86%) | 247 (59.09%) | 1158 (60.03%) |  |
| High /secondary school | 496 (21.13%) | 83 (19.86%) | 413 (21.41%) |  |
| Post-secondary education | 261 (11.12%) | 48 (11.48%) | 213 (11.04%) |  |
| Master's degree or higher | 2 (0.09%) | 0 (0.00%) | 2 (0.10%) |  |
| Stoke, n (%) |  |  |  | 0.792 |
| No | 2321 (98.89%) | 412 (98.56%) | 1909 (98.96%) |  |
| Yes | 25 (1.07%) | 5 (1.20%) | 20 (1.04%) |  |
| CHD, n (%) |  |  |  | 0.151 |
| No | 2331 (99.32%) | 407 (97.37%) | 1924 (99.74%) |  |
| Yes | 8 (0.34%) | 3 (0.72%) | 5 (0.26%) |  |
| Hypertension, n (%) |  |  |  | 0.910 |
| No | 1520 (64.76%) | 272 (65.07%) | 1248 (64.70%) |  |
| Yes | 827 (35.24%) | 146 (34.93%) | 681 (35.30%) |  |
| Diabetes, n (%) |  |  |  | 0.633 |
| No | 2141 (91.22%) | 378 (90.43%) | 1763 (91.39%) |  |
| Yes | 205 (8.73%) | 39 (9.33%) | 166 (8.61%) |  |
| Hyperlipidemia, n (%) |  |  |  | **<0.001** |
| No | 1241 (52.88%) | 281 (67.22%) | 960 (49.77%) |  |
| Yes | 1106 (47.12%) | 137 (32.78%) | 969 (50.23%) |  |
| Drug treatment, n (%) |  |  |  | 0.820 |
| No | 1852 (78.91%) | 340 (81.34%) | 1512 (78.38%) |  |
| Yes | 344 (14.66%) | 61 (14.59%) | 283 (14.67%) |  |
| Albuminuria, n (%) |  |  |  | 0.689 |
| No | 1880 (80.10%) | 212 (50.72%) | 1668 (86.47%) |  |
| Yes | 291 (12.40%) | 30 (7.18%) | 261 (13.53%) |  |
| Cognitive impairment, n (%) |  |  |  | 0.761 |
| No | 2168 (92.37%) | 388 (92.82%) | 1780 (92.28%) |  |
| Yes | 179 (7.63%) | 30 (7.18%) | 149 (7.72%) |  |

HR, heart rate; SBP, systolic blood pressure; DBP, diastolic blood pressure; Mean SBP, mean SBP of 36 years; BMI, body mass index; MMSE, Mini-Mental State Examination; CysC, cystatin C; hs-CRP, high-sensitivity C-reactive protein; FBG, fasting blood glucose; ALT, alanine aminotransferase; AST, aspartate aminotransferase; UA, uric acid; TC, total cholesterol; TG, triglycerides; HDL-C, high-density lipoprotein cholesterol; LDL-C, low-density lipoprotein cholesterol; eGFR, estimated glomerular filtration rate; uACR, urinary microalbumin-to-creatinine ratio. Bold p-values indicate statistically significant differences.

**Table S2** Pearsson correlation coefficient between CysC and covariates

|  | **CysC** | **MeanSBP** | **Mean BMI** | **Age** | **FBG** | **TG** | **LDL** | **eGFR** |
| --- | --- | --- | --- | --- | --- | --- | --- | --- |
| **CysC** | 1 |  |  |  |  |  |  |  |
| **MeanSBP** | 0.048 | 1 |  |  |  |  |  |  |
| **Mean BMI** | 0.05 | 0.414 | 1 |  |  |  |  |  |
| **Age** | 0.05 | 0.295 | 0.325 | 1 |  |  |  |  |
| **FBG** | -0.039 | 0.1 | 0.126 | 0.059 | 1 |  |  |  |
| **TG** | 0.046 | 0.088 | 0.124 | 0 | 0.176 | 1 |  |  |
| **LDL** | 0.002 | 0.026 | 0.008 | 0.009 | 0.032 | -0.186 | 1 |  |
| **eGFR** | -0.288 | -0.065 | -0.08 | -0.103 | 0.162 | 0.016 | -0.019 | 1 |

Mean SBP, mean SBP of 36 years; BMI, body mass index; CysC, cystatin C; FBG, fasting blood glucose; TG, triglycerides; HDL-C, high-density lipoprotein cholesterol; LDL-C, low-density lipoprotein cholesterol; eGFR, estimated glomerular filtration rate.

**Table S3** Association of CysC, covariate and cognitive impairment

| **Variable** |  | **Model 1** |  |  | **Model 2** |  |  | **Model 3** |  |
| --- | --- | --- | --- | --- | --- | --- | --- | --- | --- |
|  | **OR** | **95%CI** | ***p*-value** | **OR** | **95%CI** | ***p*-value** | **OR** | **95%CI** | ***p*-value** |
| CysC, mg/L | 3.40 | 1.95-5.95 | ***<0.001*** | 3.15 | 1.80-5.51 | ***<0.001*** | 3.90 | 2.12-7.31 | ***<0.001*** |
| CysC (z-score) | 1.43 | 1.22-1.68 | ***<0.001*** | 1.40 | 1.19-1.64 | ***<0.001*** | 1.49 | 1.24-1.79 | ***<0.001*** |
| CysC quartiles |  |  |  |  |  |  |  |  |  |
| Q1 | 1.00 (Ref) | - | - | 1.00 (Ref) | - | - | 1.00 (Ref) | - | - |
| Q2 | 0.76 | 0.44-1.31 | 0.332 | 0.76 | 0.44-1.32 | 0.334 | 0.78 | 0.44-1.35 | 0.368 |
| Q3 | 1.37 | 0.85-2.22 | 0.201 | 1.34 | 0.83-2.19 | 0.232 | 1.45 | 0.89-2.39 | 0.142 |
| Q4 | 1.67 | 1.06-2.68 | ***0.028*** | 1.56 | 0.98-2.50 | 0.062 | 1.79 | 1.09-2.98 | ***0.022*** |
| MeanSBP, mmHg | 1.02 | 1.01-1.04 | ***0.002*** | 1.01 | 1.00-1.03 | 0.115 | 1.01 | 1.00-1.03 | 0.121 |
| Married status, n (%) |  |  |  |  |  |  |  |  |  |
| Unmarried | 1.00 (Ref) | - | - | 1.00 (Ref) | - | - | 1.00 (Ref) | - | - |
| Married | 0.47 | 0.12-3.04 | 0.325 | 0.49 | 0.13-3.25 | 0.366 | 0.50 | 0.13-3.30 | 0.377 |
| Divorced | 0.09 | 0.00-1.02 | 0.058 | 0.10 | 0.00-1.19 | 0.076 | 0.11 | 0.00-1.22 | 0.080 |
| Widowed | 0.85 | 0.09-8.02 | 0.877 | 0.50 | 0.05-5.22 | 0.544 | 0.55 | 0.05-5.88 | 0.605 |
| Mean BMI, kg/m² | 1.08 | 1.01-1.15 | ***0.020*** | 1.01 | 0.93-1.08 | 0.876 | 1.01 | 0.93-1.09 | 0.841 |
| Age, years | 1.14 | 1.08-1.19 | ***<0.001*** | 1.12 | 1.07-1.18 | ***<0.001*** | 1.12 | 1.06-1.18 | ***<0.001*** |
| Physical activities, n (%) |  |  |  |  |  |  |  |  |  |
| No activity | 1.00 (Ref) | - | - | 1.00 (Ref) | - | - | 1.00 (Ref) | - | - |
| Mild | 1.10 | 0.60-2.15 | 0.764 | 1.06 | 0.58-2.09 | 0.856 | 1.06 | 0.57-2.08 | 0.866 |
| Moderate | 1.71 | 0.95-3.31 | 0.088 | 1.62 | 0.89-3.15 | 0.132 | 1.62 | 0.89-3.16 | 0.133 |
| Vigorous | 2.44 | 1.25-5.00 | ***0.011*** | 2.13 | 1.07-4.40 | ***0.035*** | 2.18 | 1.09-4.57 | ***0.032*** |
| Smoking | 1.02 | 0.72-1.44 | 0.895 | 0.98 | 0.69-1.38 | 0.895 | 1.11 | 0.75-1.63 | 0.609 |
| Drinking | 1.21 | 0.18-4.55 | 0.810 | 1.06 | 0.16-3.93 | 0.937 | 1.06 | 0.57-2.08 | 0.868 |
| FBG, mmol/L | 1.00 | 0.91-1.08 | 0.925 | 0.98 | 0.89-1.07 | 0.726 | 0.97 | 0.88-1.06 | 0.601 |
| TG, mmol/L | 1.05 | 0.95-1.14 | 0.265 | 1.05 | 0.94-1.14 | 0.321 | 1.06 | 0.96-1.16 | 0.214 |
| LDL, mmol/L | 1.03 | 0.80-1.32 | 0.802 | 1.02 | 0.80-1.30 | 0.852 | 1.10 | 0.85-1.41 | 0.456 |
| eGFR, mL/min/1.73m² | 1.00 | 0.99-1.01 | 0.854 | 1.00 | 0.99-1.01 | 0.665 | 1.00 | 0.99-1.01 | 0.863 |
| CHD | 1.10 | 0.85-1.41 | 0.458 | 1.09 | 0.85-1.40 | 0.481 | 1.10 | 0.75-1.61 | 0.623 |
| Stroke | 1.33 | 0.21-4.67 | 0.703 | 1.15 | 0.18-4.09 | 0.853 | 1.21 | 0.18-4.58 | 0.804 |

CysC, cystatin C; OR, Odds ratio; CI, confidence interval. Model 1: univarate. Model 2: adjusted for Gender, age, mean BMI, mean SBP. Model 3: Model 2 + marital status, physical activities, smoking habits, alcohol consumption, stroke, CHD, fasting blood glucose, triglyceride, LDL-C and eGFR. Bold p-values indicate statistically significant differences.

Model equations: Model 1, logit(P)=β0+β1×*X*CysC; Model 2, logit(*P*)=*β*0+*β*1×*X*CysC+*β*2×Gender+*β*3×Age+*β*4×MeanBMI+*β*5×MeanSBP; Model 3, logit(*P*)=*β*0+*β*1×*X*CysC+*β*2×Gender+*β*3×Age+*β*4×MeanBMI+*β*5×MeanSBP+*β*6×Marital Status+*β*7×Physical Activity+*β*8×Smoking+*β*9×Alcohol+*β*10×Stroke+*β*11×CHD+*β*12×FBG+*β*13×TG+*β*14×LDL_C+*β*15×eGFR.

**Table S4** Association of CysC and cognitive impairment excluded the covariate of eGFR

| **Variable** | **OR** | **95%CI** | ***p*-value** |
| --- | --- | --- | --- |
| CysC, mg/L | 3.49 | 1.95-6.30 | ***<0.001*** |
| CysC (z-score) | 1.44 | 1.21-1.71 | ***<0.001*** |
| CysC quartiles |  |  |  |
| Q1 | 1.00 (Ref) | - | - |
| Q2 | 0.76 | 0.43-1.31 | 0.319 |
| Q3 | 1.40 | 0.86-2.29 | 0.178 |
| Q4 | 1.67 | 1.04-2.72 | ***0.035*** |

CysC, cystatin C; OR, Odds ratio; CI, covariate: Gender, age, mean BMI, mean SBP, marital status, physical activities, smoking habits, alcohol consumption, history of stroke, history of coronary heart disease, fasting blood glucose, triglyceride and LDL-C. Bold p-values indicate statistically significant differences.

**Table S5.** Characteristics of participants grouped by albuminuria

| Variables | Total (n = 1929) | Non-albuminuria (n = 1668) | With albuminuria (n = 261) | ***p*-value** |
| --- | --- | --- | --- | --- |
| Age, years | 49 (45-50) | 49 (45-50) | 49 (46-50) | 0.329 |
| SBP, mmHg | 125 (114.5-136.5) | 124 (114-134.5) | 135.25 (123.5-149) | **<0.001** |
| DBP, mmHg | 83 (75-91) | 82 (74.5-89.5) | 89 (79.88-99.12) | **<0.001** |
| HR, beats/min | 77 (70.33-85) | 77 (70-84.67) | 79 (72.67-88.33) | **<0.001** |
| MeanSBP, mmHg | 116.43 (109.9-124.09) | 115.79 (109.42-122.97) | 120.89 (114.39-130.73) | **<0.001** |
| Mean BMI, kg/m² | 21.38 (19.8-22.86) | 21.29 (19.74-22.77) | 22.07 (20.69-23.54) | **<0.001** |
| CysC, mg/L | 1.03 (0.88-1.19) | 1.03 (0.88-1.19) | 1.04 (0.88-1.25) | 0.112 |
| Fasting insulin, mU/L | 14.67 (11.75-18.82) | 14.38 (11.52-18.27) | 16.78 (13.11-21.93) | **<0.001** |
| hs-CRP, mg/L | 0.68 (0.33-1.49) | 0.64 (0.32-1.41) | 0.98 (0.43-2.05) | **<0.001** |
| FBG, mmol/L | 5.31 (4.95-5.78) | 5.28 (4.93-5.73) | 5.67 (5.07-6.5) | **<0.001** |
| Serum Cre, μmol/L | 73.3 (64.6-82.1) | 73.2 (64.9-81.82) | 73.7 (62.8-84) | 0.594 |
| Serum UA, μmol/L | 282 (232.5-337.6) | 279.6 (230.78-334.38) | 294.2 (243.6-351.4) | **0.003** |
| TC, mmol/L | 4.73 (4.24-5.3) | 4.73 (4.24-5.27) | 4.76 (4.24-5.45) | 0.112 |
| TG, mmol/L | 1.57 (1.12-2.25) | 1.52 (1.09-2.17) | 1.89 (1.31-2.89) | **<0.001** |
| HDL, mmol/L | 1.13 (0.98-1.31) | 1.14 (0.98-1.32) | 1.08 (0.94-1.24) | **0.001** |
| LDL, mmol/L | 2.63 (2.23-3.05) | 2.63 (2.24-3.05) | 2.62 (2.2-3.06) | 0.852 |
| Lp(a), mg/L | 160 (91-272) | 157.5 (90-265.25) | 182 (94-299) | 0.146 |
| MMSE score | 28 (26-29) | 28 (26-29) | 28 (26-29) | 0.113 |
| eGFR, mL/min/1.73m² | 99.5 (90.9-110) | 99.4 (91.18-109) | 100 (87.8-112) | 0.872 |
| uACR, mg/g | 4.66 (1.79-14.02) | 3.67 (1.57-8.76) | 58.26 (37.85-125.23) | **<0.001** |
| Gender |  |  |  | 0.641 |
| Female | 891 (46.19%) | 774 (46.40%) | 117 (44.83%) |  |
| Male | 1038 (53.81%) | 894 (53.60%) | 144 (55.17%) |  |
| Smoking, n (%) |  |  |  | 0.277 |
| No | 1162 (60.24%) | 1013 (60.73%) | 149 (57.09%) |  |
| Yes | 767 (39.76%) | 655 (39.27%) | 112 (42.91%) |  |
| Drinking, n (%) |  |  |  | 0.705 |
| No | 1423 (73.77%) | 1233 (73.92%) | 190 (72.80%) |  |
| Yes | 506 (26.23%) | 435 (26.08%) | 71 (27.20%) |  |
| Married status, n (%) |  |  |  | 0.761 |
| Unmarried | 13 (0.67%) | 11 (0.66%) | 2 (0.77%) |  |
| Married | 1839 (95.33%) | 1592 (95.44%) | 247 (94.64%) |  |
| Divorced | 62 (3.21%) | 53 (3.18%) | 9 (3.45%) |  |
| Widowed | 15 (0.78%) | 12 (0.72%) | 3 (1.15%) |  |
| Physical activities, n (%) |  |  |  | 0.459 |
| No activity | 237 (12.29%) | 204 (12.23%) | 33 (12.64%) |  |
| Mild | 799 (41.42%) | 680 (40.77%) | 119 (45.59%) |  |
| Moderate | 675 (34.99%) | 593 (35.55%) | 82 (31.42%) |  |
| Vigorous | 218 (11.30%) | 191 (11.45%) | 27 (10.34%) |  |
| Education, n (%) |  |  |  | **0.007** |
| Illiteracy | 1 (0.05%) | 0 (0.00%) | 1 (0.38%) |  |
| Primary school | 142 (7.36%) | 117 (7.01%) | 25 (9.58%) |  |
| Junior high school | 1158 (60.03%) | 1001 (60.01%) | 157 (60.15%) |  |
| High /secondary school | 413 (21.41%) | 365 (21.88%) | 48 (18.39%) |  |
| Post-secondary education | 213 (11.04%) | 185 (11.09%) | 28 (10.73%) |  |
| Master's degree or higher | 2 (0.10%) | 0 (0.00%) | 2 (0.77%) |  |
| Stoke, n (%) |  |  |  | 0.335 |
| No | 1909 (98.96%) | 1652 (99.04%) | 257 (98.47%) |  |
| Yes | 20 (1.04%) | 16 (0.96%) | 4 (1.53%) |  |
| CHD, n (%) |  |  |  | 1.000 |
| No | 1924 (99.74%) | 1663 (99.70%) | 261 (100.00%) |  |
| Yes | 5 (0.26%) | 5 (0.30%) | 0 (0.00%) |  |
| Hypertension, n (%) |  |  |  | **<0.001** |
| No | 1248 (64.70%) | 1142 (68.47%) | 106 (40.61%) |  |
| Yes | 681 (35.30%) | 526 (31.53%) | 155 (59.39%) |  |
| Diabetes, n (%) |  |  |  | **<0.001** |
| No | 1763 (91.39%) | 1560 (93.53%) | 203 (77.78%) |  |
| Yes | 166 (8.61%) | 108 (6.47%) | 58 (22.22%) |  |
| Hyperlipidemia, n (%) |  |  |  | **0.009** |
| No | 960 (49.77%) | 850 (50.96%) | 110 (42.15%) |  |
| Yes | 969 (50.23%) | 818 (49.04%) | 151 (57.85%) |  |
| Drug treatment, n (%) |  |  |  | **<0.001** |
| No | 1646 (85.33%) | 1472 (88.25%) | 174 (66.67%) |  |
| Yes | 283 (14.67%) | 196 (11.75%) | 87 (33.33%) |  |
| Cognitive impairment, n (%) |  |  |  | 0.104 |
| No | 1780 (92.28%) | 1546 (92.69%) | 234 (89.66%) |  |
| Yes | 149 (7.72%) | 122 (7.31%) | 27 (10.34%) |  |

HR, heart rate; SBP, systolic blood pressure; DBP, diastolic blood pressure; Mean SBP, mean SBP of 36 years; BMI, body mass index; MMSE, Mini-Mental State Examination; CysC, cystatin C; hs-CRP, high-sensitivity C-reactive protein; FBG, fasting blood glucose; Cre, Creatinine; UA, uric acid; TC, total cholesterol; TG, triglycerides; HDL-C, high-density lipoprotein cholesterol; LDL-C, low-density lipoprotein cholesterol; eGFR, estimated glomerular filtration rate; uACR, urinary microalbumin-to-creatinine ratio. Bold p-values indicate statistically significant differences.

**Table S6.** Characteristics of participants grouped by drug treatment

| Variables | Total (n = 1929) | Non-drug (n = 1646) | With drug (n = 283) | ***p*-value** |
| --- | --- | --- | --- | --- |
| Age, years | 49 (45-50) | 49 (45-50) | 49 (46-51) | **0.011** |
| SBP, mmHg | 125 (114.5-136.5) | 123.5 (114-134.5) | 134 (124.38-147.62) | **<0.001** |
| DBP, mmHg | 83 (75-91) | 81.5 (74.5-89.5) | 90 (81.5-97.62) | **<0.001** |
| HR, beats/min | 77 (70.33-85) | 76.67 (70-84.67) | 80 (72.67-88.33) | **<0.001** |
| MeanSBP, mmHg | 116.43 (109.9-124.09) | 115.35 (109.16-122.18) | 125.52 (117.05-132.5) | **<0.001** |
| Mean BMI, kg/m² | 21.38 (19.8-22.86) | 21.25 (19.65-22.76) | 22.04 (20.82-23.64) | **<0.001** |
| CysC, mg/L | 1.03 (0.88-1.19) | 1.03 (0.88-1.19) | 1.07 (0.9-1.25) | **0.009** |
| Fasting insulin, mU/L | 14.67 (11.75-18.82) | 14.3 (11.5-18.26) | 16.66 (13.52-21.87) | **<0.001** |
| hs-CRP, mg/L | 0.68 (0.33-1.49) | 0.63 (0.31-1.39) | 0.99 (0.45-2.08) | **<0.001** |
| FBG, mmol/L | 5.31 (4.95-5.78) | 5.28 (4.92-5.72) | 5.66 (5.13-6.78) | **<0.001** |
| Serum Cre, μmol/L | 73.3 (64.6-82.1) | 72.6 (64.4-81.8) | 76.2 (66.75-83.75) | **0.003** |
| Serum UA, μmol/L | 282 (232.5-337.6) | 278.15 (228.5-334) | 300.1 (258.6-361.15) | **<0.001** |
| TC, mmol/L | 4.73 (4.24-5.3) | 4.73 (4.24-5.29) | 4.76 (4.24-5.33) | 0.730 |
| TG, mmol/L | 1.57 (1.12-2.25) | 1.51 (1.08-2.17) | 1.89 (1.33-2.76) | **<0.001** |
| HDL, mmol/L | 1.13 (0.98-1.31) | 1.14 (0.99-1.32) | 1.04 (0.92-1.23) | **<0.001** |
| LDL, mmol/L | 2.63 (2.23-3.05) | 2.62 (2.24-3.05) | 2.68 (2.2-3.05) | 0.995 |
| Lp(a), mg/L | 160 (91-272) | 159.5 (92-273) | 165 (82.5-266) | 0.615 |
| MMSE score | 28 (26-29) | 28 (26-29) | 28 (26-29) | 0.142 |
| eGFR, mL/min/1.73m² | 99.5 (90.9-110) | 99.5 (91-110) | 99.5 (90.6-110.5) | 0.710 |
| uACR, mg/g | 4.66 (1.79-14.02) | 4.17 (1.69-11.63) | 11.27 (2.97-36.29) | **<0.001** |
| Gender |  |  |  | **<0.001** |
| Female | 891 (46.19%) | 795 (48.30%) | 96 (33.92%) |  |
| Male | 1038 (53.81%) | 851 (51.70%) | 187 (66.08%) |  |
| Smoking, n (%) |  |  |  | **0.002** |
| No | 1162 (60.24%) | 1016 (61.73%) | 146 (51.59%) |  |
| Yes | 767 (39.76%) | 630 (38.27%) | 137 (48.41%) |  |
| Drinking, n (%) |  |  |  | **0.034** |
| No | 1423 (73.77%) | 1229 (74.67%) | 194 (68.55%) |  |
| Yes | 506 (26.23%) | 417 (25.33%) | 89 (31.45%) |  |
| Married status, n (%) |  |  |  | 0.053 |
| Unmarried | 13 (0.67%) | 8 (0.49%) | 5 (1.77%) |  |
| Married | 1839 (95.33%) | 1574 (95.63%) | 265 (93.64%) |  |
| Divorced | 62 (3.21%) | 53 (3.22%) | 9 (3.18%) |  |
| Widowed | 15 (0.78%) | 11 (0.67%) | 4 (1.41%) |  |
| Physical activities, n (%) |  |  |  | 0.828 |
| No activity | 237 (12.29%) | 201 (12.21%) | 36 (12.72%) |  |
| Mild | 799 (41.42%) | 676 (41.07%) | 123 (43.46%) |  |
| Moderate | 675 (34.99%) | 580 (35.24%) | 95 (33.57%) |  |
| Vigorous | 218 (11.30%) | 189 (11.48%) | 29 (10.25%) |  |
| Education, n (%) |  |  |  | 0.308 |
| Illiteracy | 1 (0.05%) | 1 (0.06%) | 0 (0.00%) |  |
| Primary school | 142 (7.36%) | 118 (7.17%) | 24 (8.48%) |  |
| Junior high school | 1158 (60.03%) | 984 (59.78%) | 174 (61.48%) |  |
| High /secondary school | 413 (21.41%) | 365 (22.17%) | 48 (16.96%) |  |
| Post-secondary education | 213 (11.04%) | 176 (10.69%) | 37 (13.07%) |  |
| Master's degree or higher | 2 (0.10%) | 2 (0.12%) | 0 (0.00%) |  |
| Stoke, n (%) |  |  |  | **<0.001** |
| No | 1909 (98.96%) | 1637 (99.45%) | 272 (96.11%) |  |
| Yes | 20 (1.04%) | 9 (0.55%) | 11 (3.89%) |  |
| CHD, n (%) |  |  |  | 0.159 |
| No | 1924 (99.74%) | 1643 (99.82%) | 281 (99.29%) |  |
| Yes | 5 (0.26%) | 3 (0.18%) | 2 (0.71%) |  |
| Hypertension, n (%) |  |  |  | **<0.001** |
| No | 1248 (64.70%) | 1188 (72.17%) | 60 (21.20%) |  |
| Yes | 681 (35.30%) | 458 (27.83%) | 223 (78.80%) |  |
| Diabetes, n (%) |  |  |  | **<0.001** |
| No | 1763 (91.39%) | 1560 (94.78%) | 203 (71.73%) |  |
| Yes | 166 (8.61%) | 86 (5.22%) | 80 (28.27%) |  |
| Hyperlipidemia, n (%) |  |  |  | **<0.001** |
| No | 960 (49.77%) | 867 (52.67%) | 93 (32.86%) |  |
| Yes | 969 (50.23%) | 779 (47.33%) | 190 (67.14%) |  |
| Albuminuria, n (%) |  |  |  | **<0.001** |
| No | 1668 (86.47%) | 1472 (89.43%) | 196 (69.26%) |  |
| Yes | 261 (13.53%) | 174 (10.57%) | 87 (30.74%) |  |
| Cognitive impairment, n (%) |  |  |  | 0.334 |
| No | 1780 (92.28%) | 1523 (92.53%) | 257 (90.81%) |  |
| Yes | 149 (7.72%) | 123 (7.47%) | 26 (9.19%) |  |

HR, heart rate; SBP, systolic blood pressure; DBP, diastolic blood pressure; Mean SBP, mean SBP of 36 years; BMI, body mass index; MMSE, Mini-Mental State Examination; CysC, cystatin C; hs-CRP, high-sensitivity C-reactive protein; FBG, fasting blood glucose; Cre, Creatinine; UA, uric acid; TC, total cholesterol; TG, triglycerides; HDL-C, high-density lipoprotein cholesterol; LDL-C, low-density lipoprotein cholesterol; eGFR, estimated glomerular filtration rate; uACR, urinary microalbumin-to-creatinine ratio. Bold p-values indicate statistically significant differences.

**Table S7.** ORs and 95% CIs of cognitive impairment without durg treatment

| **Variable** |  | **Model 1** |  |  | **Model 2** |  |  | **Model 3** |  |
| --- | --- | --- | --- | --- | --- | --- | --- | --- | --- |
|  | **OR** | **95%CI** | ***p*-value** | **OR** | **95%CI** | ***p*-value** | **OR** | **95%CI** | ***p*-value** |
| CysC, mg/L | 3.26 | 1.68-6.38 | ***<0.001*** | 3.00 | 1.54-5.87 | ***0.001*** | 3.59 | 1.75-7.67 | ***<0.001*** |
| CysC (z-score) | 1.41 | 1.16-1.72 | ***<0.001*** | 1.38 | 1.13-1.67 | ***0.001*** | 1.45 | 1.18-1.81 | ***<0.001*** |
| CysC quartiles |  |  |  |  |  |  |  |  |  |
| Q1 | 1.00 (Ref) | - | - | 1.00 (Ref) | - | - | 1.00 (Ref) | - | - |
| Q2 | 1.01 | 0.53-1.91 | 0.986 | 1.01 | 0.53-1.93 | 0.970 | 1.06 | 0.55-2.05 | 0.855 |
| Q3 | 1.50 | 0.85-2.72 | 0.165 | 1.44 | 0.81-2.62 | 0.218 | 1.56 | 0.86-2.87 | 0.146 |
| Q4 | 1.84 | 1.06-3.30 | ***0.035*** | 1.72 | 0.98-3.09 | 0.063 | 2.01 | 1.10-3.75 | ***0.024*** |

CysC, cystatin C; OR, Odds ratio; CI, confidence interval. Model 1: univarate. Model 2: adjusted for Gender, age, mean BMI, mean SBP. Model 3: Model 2 + marital status, physical activities, smoking habits, alcohol consumption, stroke, CHD, fasting blood glucose, triglyceride, LDL-C and eGFR. Bold p-values indicate statistically significant differences.

**Table S8.** Characteristics of participants grouped by gender

| Variables | Total (n = 1929) | Female (n = 891) | Male (n = 1038) | ***p*-value** |
| --- | --- | --- | --- | --- |
| Age, years | 49 (45-50) | 49 (45-50) | 49 (45-51) | 0.598 |
| SBP, mmHg | 125 (114.5-136.5) | 118.5 (109.5-131) | 129.5 (120-141) | **<0.001** |
| DBP, mmHg | 83 (75-91) | 78.5 (72-85.5) | 86.5 (79.5-94) | **<0.001** |
| HR, beats/min | 77 (70.33-85) | 76.67 (70.33-83.33) | 77.67 (70.67-86.67) | **0.023** |
| MeanSBP, mmHg | 116.43 (109.9-124.09) | 113.12 (106.99-119.46) | 119.38 (112.89-126.74) | **<0.001** |
| Mean BMI, kg/m² | 21.38 (19.8-22.86) | 21.21 (19.58-22.82) | 21.49 (20.03-22.87) | **0.004** |
| CysC, mg/L | 1.03 (0.88-1.19) | 0.98 (0.86-1.14) | 1.1 (0.92-1.25) | **<0.001** |
| Fasting insulin, mU/L | 14.67 (11.75-18.82) | 14.17 (11.37-17.87) | 15.26 (12.11-19.6) | **<0.001** |
| hs-CRP, mg/L | 0.68 (0.33-1.49) | 0.52 (0.28-1.21) | 0.84 (0.4-1.69) | **<0.001** |
| FBG, mmol/L | 5.31 (4.95-5.78) | 5.25 (4.91-5.69) | 5.37 (5-5.91) | **<0.001** |
| Serum Cre, μmol/L | 73.3 (64.6-82.1) | 64.5 (59.9-69.3) | 80.7 (75.12-87.5) | **<0.001** |
| Serum UA, μmol/L | 282 (232.5-337.6) | 237.7 (207.55-280.15) | 319.95 (277.33-368.95) | **<0.001** |
| TC, mmol/L | 4.73 (4.24-5.3) | 4.79 (4.29-5.34) | 4.69 (4.19-5.25) | **0.003** |
| TG, mmol/L | 1.57 (1.12-2.25) | 1.34 (0.98-1.91) | 1.79 (1.26-2.54) | **<0.001** |
| HDL, mmol/L | 1.13 (0.98-1.31) | 1.23 (1.09-1.42) | 1.03 (0.92-1.19) | **<0.001** |
| LDL, mmol/L | 2.63 (2.23-3.05) | 2.62 (2.23-3.03) | 2.65 (2.25-3.08) | 0.303 |
| Lp(a), mg/L | 160 (91-272) | 167 (102-282) | 152.5 (83.25-262) | **0.002** |
| MMSE score | 28 (26-29) | 28 (26-29) | 28 (26-29) | 0.905 |
| eGFR, mL/min/1.73m² | 99.5 (90.9-110) | 102 (93.4-112) | 97.75 (88.73-107) | **<0.001** |
| uACR, mg/g | 4.66 (1.79-14.02) | 4.24 (1.74-13.35) | 5.05 (1.89-15.72) | 0.084 |
| Smoking, n (%) |  |  |  | **<0.001** |
| No | 1162 (60.24%) | 864 (96.97%) | 298 (28.71%) |  |
| Yes | 767 (39.76%) | 27 (3.03%) | 740 (71.29%) |  |
| Drinking, n (%) |  |  |  | **<0.001** |
| No | 1423 (73.77%) | 850 (95.40%) | 573 (55.20%) |  |
| Yes | 506 (26.23%) | 41 (4.60%) | 465 (44.80%) |  |
| Married status, n (%) |  |  |  | 0.059 |
| Unmarried | 13 (0.67%) | 2 (0.22%) | 11 (1.06%) |  |
| Married | 1839 (95.33%) | 850 (95.40%) | 989 (95.28%) |  |
| Divorced | 62 (3.21%) | 29 (3.25%) | 33 (3.18%) |  |
| Widowed | 15 (0.78%) | 10 (1.12%) | 5 (0.48%) |  |
| Physical activities, n (%) |  |  |  | **<0.001** |
| No activity | 237 (12.29%) | 115 (12.91%) | 122 (11.75%) |  |
| Mild | 799 (41.42%) | 433 (48.60%) | 366 (35.26%) |  |
| Moderate | 675 (34.99%) | 310 (34.79%) | 365 (35.16%) |  |
| Vigorous | 218 (11.30%) | 33 (3.70%) | 185 (17.82%) |  |
| Education, n (%) |  |  |  | **<0.001** |
| Illiteracy | 1 (0.05%) | 0 (0.00%) | 1 (0.10%) |  |
| Primary school | 142 (7.36%) | 52 (5.84%) | 90 (8.67%) |  |
| Junior high school | 1158 (60.03%) | 576 (64.65%) | 582 (56.07%) |  |
| High /secondary school | 413 (21.41%) | 180 (20.20%) | 233 (22.45%) |  |
| Post-secondary education | 213 (11.04%) | 81 (9.09%) | 132 (12.72%) |  |
| Master's degree or higher | 2 (0.10%) | 2 (0.22%) | 0 (0.00%) |  |
| Stoke, n (%) |  |  |  | 0.071 |
| No | 1909 (98.96%) | 886 (99.44%) | 1023 (98.55%) |  |
| Yes | 20 (1.04%) | 5 (0.56%) | 15 (1.45%) |  |
| CHD, n (%) |  |  |  | 0.382 |
| No | 1924 (99.74%) | 890 (99.89%) | 1034 (99.61%) |  |
| Yes | 5 (0.26%) | 1 (0.11%) | 4 (0.39%) |  |
| Hypertension, n (%) |  |  |  | **<0.001** |
| No | 1248 (64.70%) | 692 (77.67%) | 556 (53.56%) |  |
| Yes | 681 (35.30%) | 199 (22.33%) | 482 (46.44%) |  |
| Diabetes, n (%) |  |  |  | **<0.001** |
| No | 1763 (91.39%) | 837 (93.94%) | 926 (89.21%) |  |
| Yes | 166 (8.61%) | 54 (6.06%) | 112 (10.79%) |  |
| Hyperlipidemia, n (%) |  |  |  | **<0.001** |
| No | 960 (49.77%) | 587 (65.88%) | 373 (35.93%) |  |
| Yes | 969 (50.23%) | 304 (34.12%) | 665 (64.07%) |  |
| Drug treatment, n (%) |  |  |  | **<0.001** |
| No | 1646 (85.33%) | 795 (89.23%) | 851 (81.98%) |  |
| Yes | 283 (14.67%) | 96 (10.77%) | 187 (18.02%) |  |
| Albuminuria, n (%) |  |  |  | 0.641 |
| No | 1668 (86.47%) | 774 (86.87%) | 894 (86.13%) |  |
| Yes | 261 (13.53%) | 117 (13.13%) | 144 (13.87%) |  |
| Cognitive impairment, n (%) |  |  |  | 0.864 |
| No | 1780 (92.28%) | 821 (92.14%) | 959 (92.39%) |  |
| Yes | 149 (7.72%) | 70 (7.86%) | 79 (7.61%) |  |

HR, heart rate; SBP, systolic blood pressure; DBP, diastolic blood pressure; Mean SBP, mean SBP of 36 years; BMI, body mass index; MMSE, Mini-Mental State Examination; CysC, cystatin C; hs-CRP, high-sensitivity C-reactive protein; FBG, fasting blood glucose; Cre, Creatinine; UA, uric acid; TC, total cholesterol; TG, triglycerides; HDL-C, high-density lipoprotein cholesterol; LDL-C, low-density lipoprotein cholesterol; eGFR, estimated glomerular filtration rate; uACR, urinary microalbumin-to-creatinine ratio. Bold p-values indicate statistically significant differences.

**Table S9.** Characteristics of participants grouped by alcohol consumption

| Variables | Total (n = 1929) | Alcohol non-consumers (n = 1423) | Alcohol consumers (n = 506) | ***p*-value** |
| --- | --- | --- | --- | --- |
| Age, years | 49 (45-50) | 49 (45-50) | 49 (45-51) | 0.923 |
| SBP, mmHg | 125 (114.5-136.5) | 123 (113-134.5) | 130 (120-142) | **<0.001** |
| DBP, mmHg | 83 (75-91) | 81 (73.5-89) | 87 (81-95.5) | **<0.001** |
| HR, beats/min | 77 (70.33-85) | 77 (70.33-84.33) | 77.67 (71.33-87.33) | **0.025** |
| MeanSBP, mmHg | 116.43 (109.9-124.09) | 115.61 (108.97-122.94) | 119.02 (112.73-127.02) | **<0.001** |
| Mean BMI, kg/m² | 21.38 (19.8-22.86) | 21.34 (19.75-22.84) | 21.48 (20.02-22.92) | 0.100 |
| CysC, mg/L | 1.03 (0.88-1.19) | 1.02 (0.87-1.18) | 1.09 (0.92-1.24) | **<0.001** |
| Fasting insulin, mU/L | 14.67 (11.75-18.82) | 14.4 (11.54-18.42) | 15.54 (12.25-19.88) | **<0.001** |
| hs-CRP, mg/L | 0.68 (0.33-1.49) | 0.62 (0.31-1.36) | 0.9 (0.41-1.77) | **<0.001** |
| FBG, mmol/L | 5.31 (4.95-5.78) | 5.29 (4.93-5.74) | 5.41 (5.02-5.96) | **<0.001** |
| Serum Cre, μmol/L | 73.3 (64.6-82.1) | 69.6 (62.8-78.9) | 80.15 (74.12-86.8) | **<0.001** |
| Serum UA, μmol/L | 282 (232.5-337.6) | 265.4 (224.25-317.55) | 323.05 (279.02-369.87) | **<0.001** |
| TC, mmol/L | 4.73 (4.24-5.3) | 4.74 (4.24-5.3) | 4.73 (4.25-5.3) | 0.730 |
| TG, mmol/L | 1.57 (1.12-2.25) | 1.45 (1.06-2.13) | 1.81 (1.32-2.57) | **<0.001** |
| HDL, mmol/L | 1.13 (0.98-1.31) | 1.15 (1-1.33) | 1.05 (0.93-1.24) | **<0.001** |
| LDL, mmol/L | 2.63 (2.23-3.05) | 2.62 (2.21-3.05) | 2.66 (2.29-3.08) | 0.123 |
| Lp(a), mg/L | 160 (91-272) | 163 (95-278) | 151.5 (81.25-259) | 0.059 |
| MMSE score | 28 (26-29) | 28 (26-29) | 28 (26-29) | 0.969 |
| eGFR, mL/min/1.73m² | 99.5 (90.9-110) | 100 (91.3-110.5) | 98.1 (89.23-107) | **<0.001** |
| uACR, mg/g | 4.66 (1.79-14.02) | 4.48 (1.75-13.46) | 5.25 (1.96-17.6) | 0.070 |
| Gender |  |  |  | **<0.001** |
| Female | 891 (46.19%) | 850 (59.73%) | 41 (8.10%) |  |
| Male | 1038 (53.81%) | 573 (40.27%) | 465 (91.90%) |  |
| Smoking, n (%) |  |  |  | **<0.001** |
| No | 1162 (60.24%) | 1032 (72.52%) | 130 (25.69%) |  |
| Yes | 767 (39.76%) | 391 (27.48%) | 376 (74.31%) |  |
| Married status, n (%) |  |  |  | 0.700 |
| Unmarried | 13 (0.67%) | 9 (0.63%) | 4 (0.79%) |  |
| Married | 1839 (95.33%) | 1356 (95.29%) | 483 (95.45%) |  |
| Divorced | 62 (3.21%) | 45 (3.16%) | 17 (3.36%) |  |
| Widowed | 15 (0.78%) | 13 (0.91%) | 2 (0.40%) |  |
| Physical activities, n (%) |  |  |  | **<0.001** |
| No activity | 237 (12.29%) | 178 (12.51%) | 59 (11.66%) |  |
| Mild | 799 (41.42%) | 619 (43.50%) | 180 (35.57%) |  |
| Moderate | 675 (34.99%) | 489 (34.36%) | 186 (36.76%) |  |
| Vigorous | 218 (11.30%) | 137 (9.63%) | 81 (16.01%) |  |
| Education, n (%) |  |  |  | 0.080 |
| Illiteracy | 1 (0.05%) | 0 (0.00%) | 1 (0.20%) |  |
| Primary school | 142 (7.36%) | 97 (6.82%) | 45 (8.89%) |  |
| Junior high school | 1158 (60.03%) | 868 (61.00%) | 290 (57.31%) |  |
| High /secondary school | 413 (21.41%) | 310 (21.78%) | 103 (20.36%) |  |
| Post-secondary education | 213 (11.04%) | 146 (10.26%) | 67 (13.24%) |  |
| Master's degree or higher | 2 (0.10%) | 2 (0.14%) | 0 (0.00%) |  |
| Stoke, n (%) |  |  |  | 0.315 |
| No | 1909 (98.96%) | 1406 (98.81%) | 503 (99.41%) |  |
| Yes | 20 (1.04%) | 17 (1.19%) | 3 (0.59%) |  |
| CHD, n (%) |  |  |  | 1.000 |
| No | 1924 (99.74%) | 1419 (99.72%) | 505 (99.80%) |  |
| Yes | 5 (0.26%) | 4 (0.28%) | 1 (0.20%) |  |
| Hypertension, n (%) |  |  |  | **<0.001** |
| No | 1248 (64.70%) | 992 (69.71%) | 256 (50.59%) |  |
| Yes | 681 (35.30%) | 431 (30.29%) | 250 (49.41%) |  |
| Diabetes, n (%) |  |  |  | 0.231 |
| No | 1763 (91.39%) | 1307 (91.85%) | 456 (90.12%) |  |
| Yes | 166 (8.61%) | 116 (8.15%) | 50 (9.88%) |  |
| Hyperlipidemia, n (%) |  |  |  | **<0.001** |
| No | 960 (49.77%) | 764 (53.69%) | 196 (38.74%) |  |
| Yes | 969 (50.23%) | 659 (46.31%) | 310 (61.26%) |  |
| Drug treatment, n (%) |  |  |  | **0.034** |
| No | 1646 (85.33%) | 1229 (86.37%) | 417 (82.41%) |  |
| Yes | 283 (14.67%) | 194 (13.63%) | 89 (17.59%) |  |
| Albuminuria, n (%) |  |  |  | 0.705 |
| No | 1668 (86.47%) | 1233 (86.65%) | 435 (85.97%) |  |
| Yes | 261 (13.53%) | 190 (13.35%) | 71 (14.03%) |  |
| Cognitive impairment, n (%) |  |  |  | **0.033** |
| No | 1780 (92.28%) | 1302 (91.50%) | 478 (94.47%) |  |
| Yes | 149 (7.72%) | 121 (8.50%) | 28 (5.53%) |  |

HR, heart rate; SBP, systolic blood pressure; DBP, diastolic blood pressure; Mean SBP, mean SBP of 36 years; BMI, body mass index; MMSE, Mini-Mental State Examination; CysC, cystatin C; hs-CRP, high-sensitivity C-reactive protein; FBG, fasting blood glucose; Cre, Creatinine; UA, uric acid; TC, total cholesterol; TG, triglycerides; HDL-C, high-density lipoprotein cholesterol; LDL-C, low-density lipoprotein cholesterol; eGFR, estimated glomerular filtration rate; uACR, urinary microalbumin-to-creatinine ratio. Bold p-values indicate statistically significant differences.

**Table S10.** Characteristics of participants grouped by hypertension

| Variables | Total (n = 1929) | Normotension (n = 1248) | Hypertension (n = 681) | ***p*-value** |
| --- | --- | --- | --- | --- |
| Age, years | 49 (45-50) | 48 (45-50) | 49 (46-51) | **<0.001** |
| SBP, mmHg | 125 (114.5-136.5) | 118 (110-125.5) | 142 (133.5-150.38) | **<0.001** |
| DBP, mmHg | 83 (75-91) | 78 (72.5-83.5) | 94 (90.5-100) | **<0.001** |
| HR, beats/min | 77 (70.33-85) | 76.67 (69.67-84) | 78.33 (71.67-87) | **<0.001** |
| MeanSBP, mmHg | 116.43 (109.9-124.09) | 112.76 (107.38-118.52) | 125.47 (117.9-132.5) | **<0.001** |
| Mean BMI, kg/m² | 21.38 (19.8-22.86) | 21.08 (19.56-22.57) | 21.91 (20.25-23.35) | **<0.001** |
| CysC, mg/L | 1.03 (0.88-1.19) | 1.02 (0.87-1.18) | 1.07 (0.91-1.23) | **<0.001** |
| Fasting insulin, mU/L | 14.67 (11.75-18.82) | 13.97 (11.3-17.5) | 16.26 (12.85-20.61) | **<0.001** |
| hs-CRP, mg/L | 0.68 (0.33-1.49) | 0.58 (0.29-1.27) | 0.9 (0.43-1.89) | **<0.001** |
| FBG, mmol/L | 5.31 (4.95-5.78) | 5.26 (4.92-5.69) | 5.42 (5.02-6.06) | **<0.001** |
| Serum Cre, μmol/L | 73.3 (64.6-82.1) | 70.5 (63.1-80.23) | 77.3 (68.8-84.1) | **<0.001** |
| Serum UA, μmol/L | 282 (232.5-337.6) | 265.6 (222.17-319.05) | 307.1 (260.5-361.7) | **<0.001** |
| TC, mmol/L | 4.73 (4.24-5.3) | 4.7 (4.23-5.26) | 4.79 (4.25-5.34) | 0.074 |
| TG, mmol/L | 1.57 (1.12-2.25) | 1.42 (1.04-2.08) | 1.82 (1.3-2.57) | **<0.001** |
| HDL, mmol/L | 1.13 (0.98-1.31) | 1.16 (1.01-1.34) | 1.07 (0.93-1.24) | **<0.001** |
| LDL, mmol/L | 2.63 (2.23-3.05) | 2.61 (2.23-3.02) | 2.69 (2.25-3.11) | 0.068 |
| Lp(a), mg/L | 160 (91-272) | 160 (95-273) | 159 (84-268) | 0.349 |
| MMSE score | 28 (26-29) | 28 (26-29) | 28 (26-29) | 0.110 |
| eGFR, mL/min/1.73m² | 99.5 (90.9-110) | 100 (91.4-110) | 98.6 (90-108) | **0.009** |
| uACR, mg/g | 4.66 (1.79-14.02) | 3.53 (1.52-9.13) | 9.24 (3.17-27.05) | **<0.001** |
| Gender |  |  |  | **<0.001** |
| Female | 891 (46.19%) | 692 (55.45%) | 199 (29.22%) |  |
| Male | 1038 (53.81%) | 556 (44.55%) | 482 (70.78%) |  |
| Smoking, n (%) |  |  |  | **<0.001** |
| No | 1162 (60.24%) | 845 (67.71%) | 317 (46.55%) |  |
| Yes | 767 (39.76%) | 403 (32.29%) | 364 (53.45%) |  |
| Drinking, n (%) |  |  |  | **<0.001** |
| No | 1423 (73.77%) | 992 (79.49%) | 431 (63.29%) |  |
| Yes | 506 (26.23%) | 256 (20.51%) | 250 (36.71%) |  |
| Married status, n (%) |  |  |  | 0.233 |
| Unmarried | 13 (0.67%) | 6 (0.48%) | 7 (1.03%) |  |
| Married | 1839 (95.33%) | 1198 (95.99%) | 641 (94.13%) |  |
| Divorced | 62 (3.21%) | 36 (2.88%) | 26 (3.82%) |  |
| Widowed | 15 (0.78%) | 8 (0.64%) | 7 (1.03%) |  |
| Physical activities, n (%) |  |  |  | 0.157 |
| No activity | 237 (12.29%) | 149 (11.94%) | 88 (12.92%) |  |
| Mild | 799 (41.42%) | 519 (41.59%) | 280 (41.12%) |  |
| Moderate | 675 (34.99%) | 452 (36.22%) | 223 (32.75%) |  |
| Vigorous | 218 (11.30%) | 128 (10.26%) | 90 (13.22%) |  |
| Education, n (%) |  |  |  | 0.191 |
| Illiteracy | 1 (0.05%) | 0 (0.00%) | 1 (0.15%) |  |
| Primary school | 142 (7.36%) | 84 (6.73%) | 58 (8.52%) |  |
| Junior high school | 1158 (60.03%) | 742 (59.46%) | 416 (61.09%) |  |
| High /secondary school | 413 (21.41%) | 273 (21.88%) | 140 (20.56%) |  |
| Post-secondary education | 213 (11.04%) | 148 (11.86%) | 65 (9.54%) |  |
| Master's degree or higher | 2 (0.10%) | 1 (0.08%) | 1 (0.15%) |  |
| Stoke, n (%) |  |  |  | **0.002** |
| No | 1909 (98.96%) | 1242 (99.52%) | 667 (97.94%) |  |
| Yes | 20 (1.04%) | 6 (0.48%) | 14 (2.06%) |  |
| CHD, n (%) |  |  |  | 0.353 |
| No | 1924 (99.74%) | 1246 (99.84%) | 678 (99.56%) |  |
| Yes | 5 (0.26%) | 2 (0.16%) | 3 (0.44%) |  |
| Diabetes, n (%) |  |  |  | **<0.001** |
| No | 1763 (91.39%) | 1168 (93.59%) | 595 (87.37%) |  |
| Yes | 166 (8.61%) | 80 (6.41%) | 86 (12.63%) |  |
| Hyperlipidemia, n (%) |  |  |  | **<0.001** |
| No | 960 (49.77%) | 682 (54.65%) | 278 (40.82%) |  |
| Yes | 969 (50.23%) | 566 (45.35%) | 403 (59.18%) |  |
| Drug treatment, n (%) |  |  |  | **<0.001** |
| No | 1646 (85.33%) | 1188 (95.19%) | 458 (67.25%) |  |
| Yes | 283 (14.67%) | 60 (4.81%) | 223 (32.75%) |  |
| Albuminuria, n (%) |  |  |  | **<0.001** |
| No | 1668 (86.47%) | 1142 (91.51%) | 526 (77.24%) |  |
| Yes | 261 (13.53%) | 106 (8.49%) | 155 (22.76%) |  |
| Cognitive impairment, n (%) |  |  |  | 0.108 |
| No | 1780 (92.28%) | 1161 (93.03%) | 619 (90.90%) |  |
| Yes | 149 (7.72%) | 87 (6.97%) | 62 (9.10%) |  |

HR, heart rate; SBP, systolic blood pressure; DBP, diastolic blood pressure; Mean SBP, mean SBP of 36 years; BMI, body mass index; MMSE, Mini-Mental State Examination; CysC, cystatin C; hs-CRP, high-sensitivity C-reactive protein; FBG, fasting blood glucose; ALT, alanine aminotransferase; AST, aspartate aminotransferase; UA, uric acid; TC, total cholesterol; TG, triglycerides; HDL-C, high-density lipoprotein cholesterol; LDL-C, low-density lipoprotein cholesterol; eGFR, estimated glomerular filtration rate; uACR, urinary microalbumin-to-creatinine ratio. Bold p-values indicate statistically significant differences.
